# Nitrogen-rich metal–organic framework of nickel(ii) as a highly efficient and reusable catalyst for the synthesis of cyclic carbonates at ambient pressure of CO_2_†

**DOI:** 10.1039/d4ra08614g

**Published:** 2025-03-06

**Authors:** Reza Erfani-Ghorbani, Hossein Eshghi, Ali Shiri

**Affiliations:** a Department of Chemistry, Faculty of Science, Ferdowsi University of Mashhad Mashhad Iran heshghi@um.ac.ir

## Abstract

Nitrogen-rich metal organic frameworks (MOFs) structures have a great potential for the chemical fixation of CO_2_. In this direction, we have utilized the highly efficient nitrogen-rich dual linker MOF of nickel(ii) as a heterogeneous catalyst in solvent-free chemical fixation of CO_2_ into cyclic carbonates at ambient pressure. In this present work, nitrogen-rich nickel-MOF, Ni-ImzAdn, was synthesized from imidazole and adenine as efficient nitrogen-rich linkers under hydrothermal conditions (Imz = Imidazole and Adn = Adenine). The Ni-ImzAdn was characterized thoroughly *via* various physicochemical analyses such as FT-IR, SEM, EDX, EDX-mapping, XRD, ICP-OES, BET, BJH, TG-DTA, CO_2_-TPD, and NH_3_-TPD. Ni-ImzAdn with adequate free nitrogen sites exhibit high catalytic activity in the cycloaddition of CO_2_ with styrene oxide (93% yield) at solvent-free and ambient pressure. The high activity of Ni–ImzAdn was attributed to the synergistic effect of strong Lewis acid and strong Lewis base sites on the catalyst, which were acquired by CO_2_ and NH_3_-TPD respectively. In addition, the MOF catalyst was presented as highly recyclable without significant loss of activity after six reaction cycles and low metal ion leaching (analyzed by (ICP-OES)). Thermogravimetry-differential thermal analysis (TG-DTA) showed the MOF catalyst had high thermal stability up to 318 °C.

## Abstract 2

Nitrogen-rich metal organic frameworks (MOFs) structures have a great potential for the chemical fixation of CO_2_.

## Graphical abstract

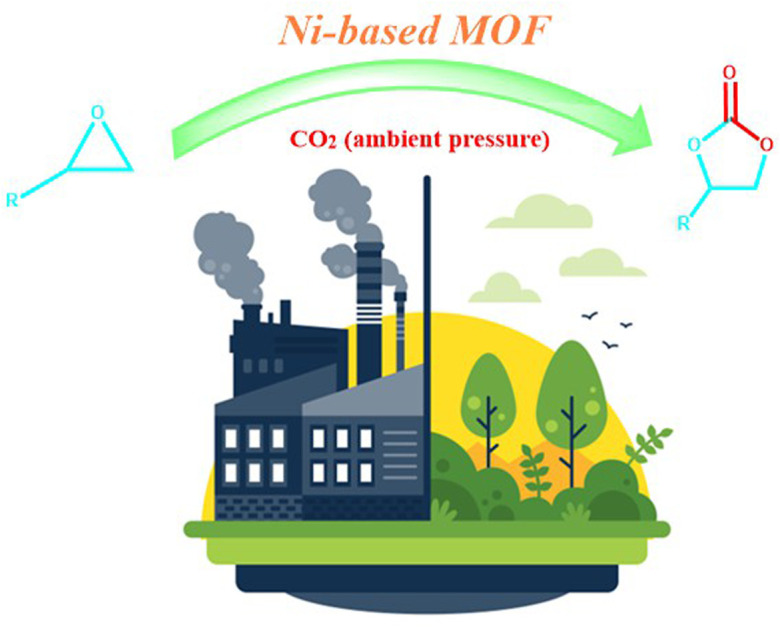

## 1. Introduction

The increasing emission of carbon dioxide (CO_2_) into the atmosphere is a critical issue contributing to global warming. As a result, extensive research has been dedicated to utilizing CO_2_ as a feedstock for synthesizing valuable materials.^1–5^ The synthesis of cyclic carbonates is a 100% atom–economic reaction, a green process, and the most efficient artificial CO_2_ fixation.^6,7^ From the product viewpoint, cyclic carbonates have diverse applications, including their use as polar aprotic solvents, electrolytes in secondary batteries, and pharmaceutical intermediates.^8–10^ Due to the thermodynamic stability of CO_2_ (standard heat of formation of Δ*H*_f_ = −394 kJ mol^−1^ and a standard Gibbs energy of Δ*G*_f_ = −395 kJ mol^−1^) percentage conversion of cyclic carbonate is highly reliant on catalysts that can significantly lower the activation barrier for the reaction.^11^ Various heterogeneous catalysts with multiple active sites have been extensively studied for the cycloaddition of CO_2_ and epoxides to synthesize cyclic carbonates.^12–18^ Among them, MOFs are the class of materials that contain many inherently excellent properties, such as high surface area, structural diversity and rich functionalities.^19,20^ As a result, MOFs have gained significant attention as promising materials for the widespread utilization of CO_2_ in producing valuable chemicals.^21–23^ Extensive studies have been done on the modification of the nitrogen-rich metal–organic frameworks with Lewis acidic open metal sites and ligands containing electron-rich nitrogen atoms for CO_2_ fixation.^24–28^

**Fig. 1:**
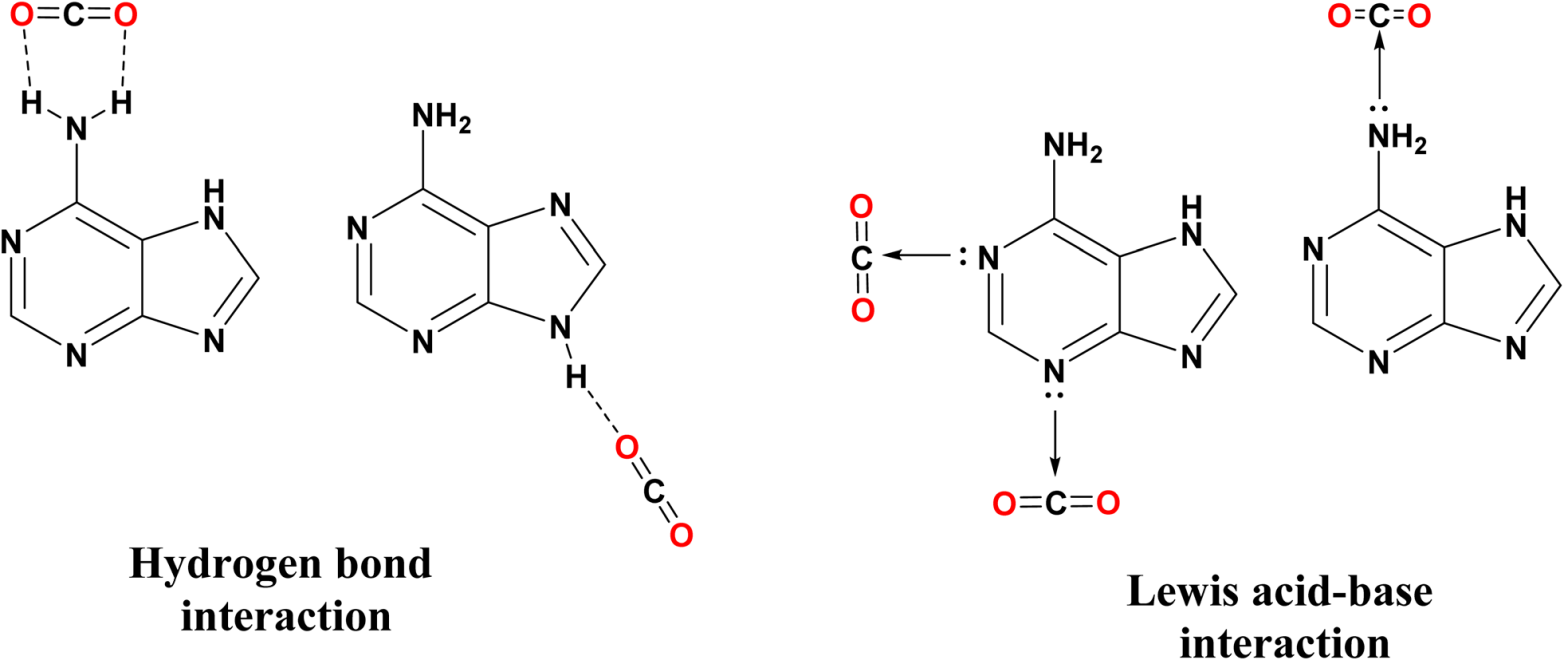
Binding interactions between nitrogen sites of adenine and CO_2_.

Bio-organic ligands are alternatives to toxic organic building blocks due to their biocompatibility, strong metal–ligand coordination, and CO_2_ capture capability.^29–31^ Adenine is a suitable ligand because of its multiple uncoordinated free nitrogen sites (consisting of one exocyclic amino-N atom and four heterocyclic N atoms) which could facilitate CO_2_ adsorption by Lewis acid-base and hydrogen bond interaction between CO_2_ and adenine ligands in chemical fixation of CO_2_ (Fig. 1).^32–35^

**Fig. 2:**
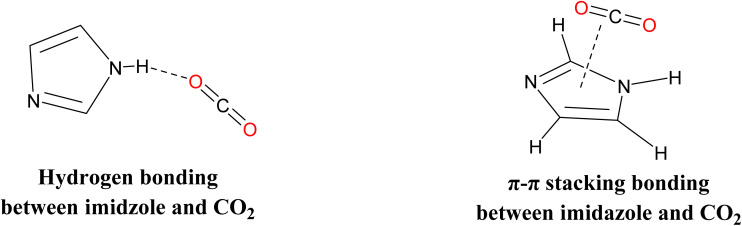
Different types of interaction for CO_2_ adsorption by imidazole.

Imidazole is an N-containing heterocyclic ring that contains multi-binding sites for high CO_2_ adsorption capacity. This is due to strong hydrogen bonds –NH group and strong intramolecular dispersive π–π stacking bonding between imidazole and CO_2_ (the π–π stacking conformation is 15.1 kJ mol^−1^) (Fig. 2).^36,37^

Because of the aforesaid discussion, a suitable MOF-based catalysts for CO_2_ fixation under mild conditions should have a high density of CO_2_-philic sites such as nitrogen functionalities and Lewis acid sites (metal nodes), which combine high CO_2_ adsorption and epoxide activation. With this purpose, we have utilized Ni–ImzAdn MOF for CO_2_ cycloaddition with epoxide into cyclic carbonates under solvent-free conditions.

## 2. Experimental

### 2.1 Materials and methods

Epoxides (purity >98%), tetrabutylammonium bromide (TBAB, >98%), imidazole, adenine, and Ni(NO_3_)_2_·6H_2_O were procured from Sigma-Aldrich. Carbon dioxide (CO_2_ > 80%) was used for the synthesis of cyclic carbonates. Organic solvents such as methanol, chloroform, and ethanol were purchased from Merck Chemical Industries. The FT-IR spectrums were measured by the Thermo Nicolet Avatar 370. Leaching of metal was measured by the ICP-OES (inductively coupled plasma-optical emission spectrometry) method using the AMETEK (ARCOS FHE12) model Spectro Arcos-76004555 plasma. NMR spectra were recorded on a Bruker Avance III 300 MHz spectrometer. Field emission scanning electron microscopy (FE SEM; MIRA3 TESCAN) equipped with an energy dispersive X-ray spectroscopy (EDX) was used to analyze the morphology and dimensions of the samples. CO_2_/NH_3_ temperature programmed desorption (CO_2_/NH_3_-TPD) was performed on a NanoSROD (made by Sensiran Co., Iran) instrument. N_2_ adsorption–desorption isotherms were measured by using a GasSorb II (made by Toos Nano Co., Iran) instrument. XRD patterns were collected using a high-resolution diffractometer (Explorer, GNR Analytical Instruments, Italy). Thermogravimetry-differential thermal analysis (TG-DTA) was carried out using a Bahr STA-503 instrument. The samples (20.4 mg) were placed in aluminum pans and heated up to 800 °C at a rate of 5 °C min^−1^ under an air stream.

### 2.2 Synthesis of the nickel-imidazole adenine MOF (Ni–ImzAdn)

The Ni–ImzAdn catalysis was prepared according to the literature with a slight modification.^38^ To be specific, 3 mmol of [Ni(NO_3_)_2_·6H_2_O] and 12 mmol of the organic ligands (imidazole (50%) + adenine (50%)) were dissolved in ethanol. The homogeneous solution mixture was transferred into a 100 mL Teflon-lined stainless steel autoclave and held at 180 °C for 10 h to achieve nucleation for the growth of the semi-crystalline MOF. The reaction mixture was cooled to room temperature and spring green-colored material was separated by filtration. The solid sample was further washed with methanol and chloroform for purification. The product was dried at 80 °C for 18 h to remove the residual solvent.

### 2.3 General synthesis of cyclic carbonates

The CO_2_ and epoxide cycloaddition reactions were performed in a round bottom flask fitted with a Teflon stopcock flow control adaptor, a CO_2_ balloon, and a magnetic stirrer. The experiment was performed using CO_2_ cycloaddition with styrene oxide (SO) as a model reaction. In a typical experiment, the solvent-free condition was carried out by mixing epoxide (1.50 mmol), catalyst (0.020 g), and co-catalyst TBAB (0.15 mmol, 0.050 g) in a round-bottom flask, which was connected to a CO_2_ balloon. Then, the reaction was stirred at 100 °C for 8 h. After the completion of the chemical reaction (monitored by thin-layer chromatography (TLC)), the reactor was cooled to room temperature, and the catalyst was removed through centrifugation strategies. The resulting crude product was purified by column chromatography using ethyl acetate : *n*-hexane (4 : 10) to get the pure product (0.229 g, 1.39 mmol, 93% yield). The catalyst was washed with methanol (3 × 10 mL), chloroform (3 × 10 mL), and dried at 80 °C overnight for the next run.

## 3. Results and discussion

### 3.1 Catalyst characterizations

**Fig. 3:**
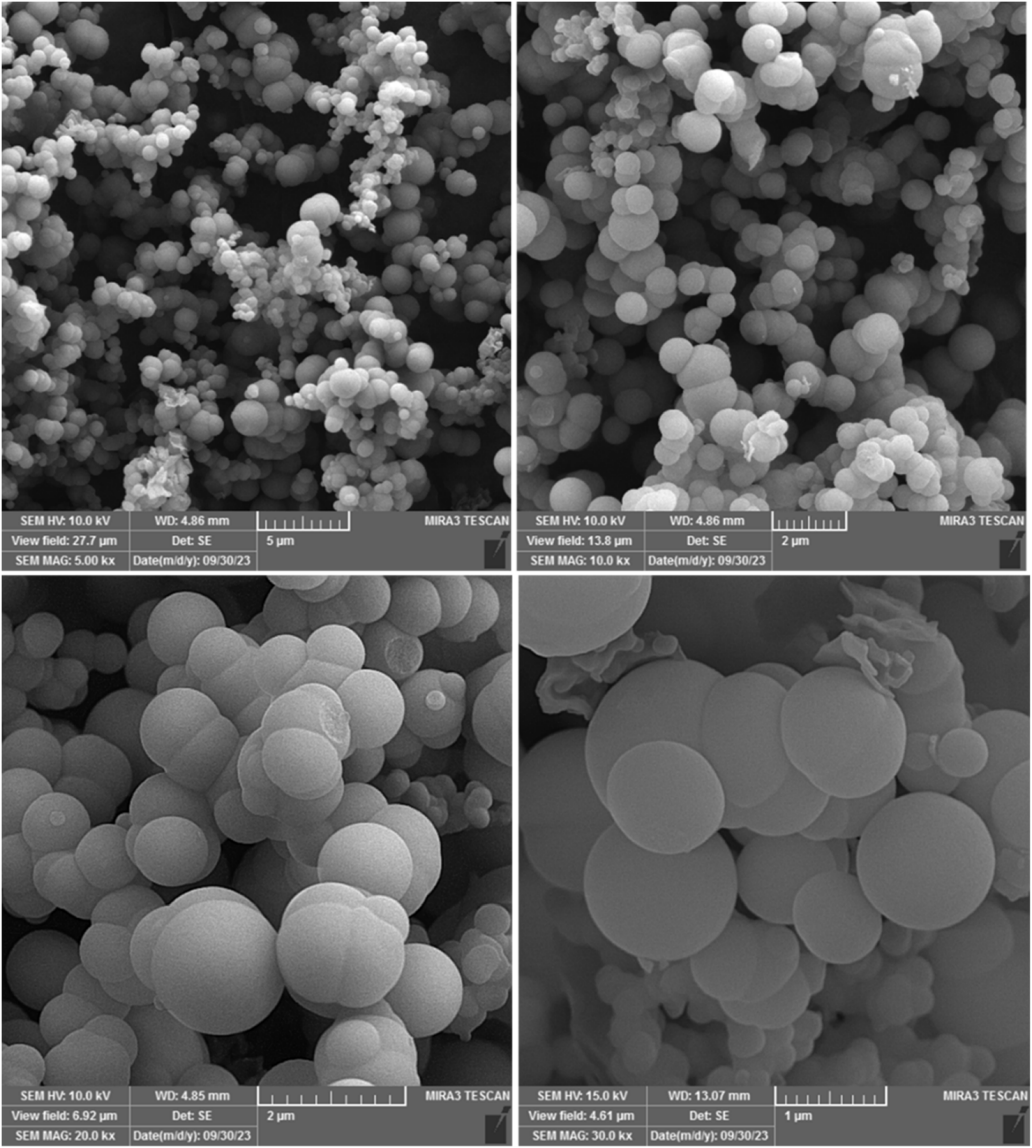
SEM images of Ni–ImzAdn MOF.

The FT-IR spectroscopy of Ni–ImzAdn MOF was measured, as illustrated in Fig. S1, ESI.† Absorption bands in the region 3201–3465 cm^−1^ are attributed to the NH and NH_2_ stretching vibrations of adenine and imidazole. The MOF catalyst shows a strong band for C=N double bond group that appears in the range of 1596–1643 cm^−1^ for adenine. The presence of peaks at 1205, 1335 and 1074 cm^−1^ can be attributed to the scissoring-wagging-stretching vibration of the imidazole molecule.^38^ Further, the O–Ni–O bond had a peak at 658 cm^−1^.^38,39^ The X-ray diffraction (XRD) shows the formation of the MOF materials and crystalline nature of the Ni–ImzAdn (Fig. S2, ESI†). The Ni–O peaks at 2*θ* = 10, 25, 16, and 33 are attributed to the formation of the nickel oxide clusters in Ni–ImzAdn MOF.^38^ The peak detected around 2*θ* = 13.1 is characteristic of adenine.^38^ Furthermore, the XRD pattern of the Ni–ImzAdn MOF shows that the peaks at 2*θ* = 8, 23, 26, and 30 correspond to the MOF formation.^38^ Moreover, due to factors including the nucleation of MOF materials, specific synthetic methods, solvothermal synthesis, temperature, solvents, reagent concentration of the Ni–ImzAdn MOF, the catalyst resulted into a semi-amorphous MOF.^40,41^ The synthesized framework in this study exhibits significant broadening in the region above 10 due to its amorphous structure or fine crystalline particles, making single-crystal analysis impossible. However, the main characteristic of the synthesized framework shows two sharp peaks at 2*θ* = 8 and 23. The surface morphology of the Ni–ImzAdn was performed using scanning electron microscopy (SEM), energy dispersive X-ray (EDX) analysis, and element mapping technique. The SEM revealed a spherical shape and smooth structure of Ni–ImzAdn (Fig. 3). The chemical constituents and the purity of the Ni–ImzAdn were done by energy dispersive X-ray (EDX) analysis and elemental mapping, where peaks for nickel, oxygen, nitrogen and carbon, were observed with ratios of 17.49, 13.55, 40.27, and 28.69, respectively, as shown in Fig. S3(b and c), ESI.† The nickel and other elements were dispersed uniformly on the catalyst surface.

**Fig. 4:**
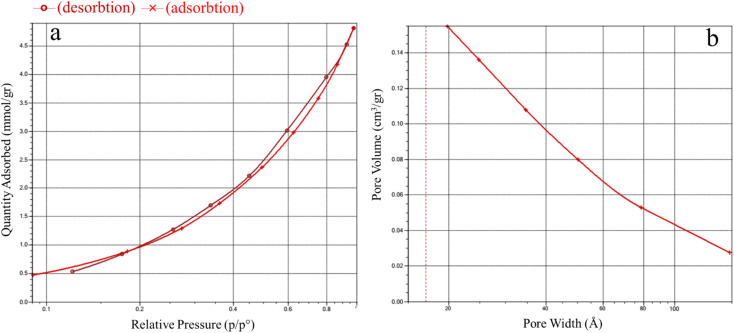
BET adsorption–desorption isotherm (a) and BJH pore-size distribution (b) of Ni–ImzAdn MOF.

Brunauer–Emmett–Teller (BET) and Barrett–Joyner–Halenda (BJH) are the most important factors affecting the catalytic activity.^40,42^ The BET analysis results for Ni–ImzAdn exhibited the type II adsorption–desorption isotherm as per the International Union of Pure and Applied Chemistry (IUPAC) classification (Fig. 4(a)).^38^ In the range of *P*/*P*_o_ (0.1–1), Ni–ImzAdn showed a slight increase in the volume of nitrogen adsorption, which indicates the presence of micropores in the structure. Moreover, the specific surface area and pore volume are approximately 161.8486 m^2^ g^−1^ and 0.146227 cm^3^ g^−1^, respectively. Additionally, the BJH isotherm in Fig. 4(b) clearly shows pores at approximately 1.9 nm that correspond to the micropores MOF. The pore size distribution and the surface functionality of MOF are the key issues for the CO_2_ adsorption capacities.^43,44^ Therefore, the microporous Ni–ImzAdn MOF with polar uncoordinated N-rich sites can enhance the adsorption of CO_2_.

**Fig. 5:**
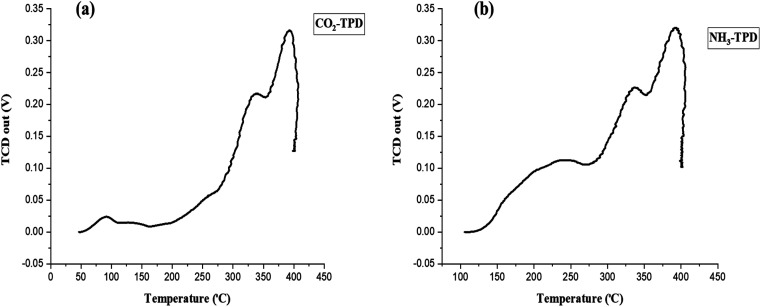
CO_2_-TPD (a) and NH_3_-TPD (b) of Ni–ImzAdn MOF.

The MOF-catalyzed epoxide-CO_2_ cycloaddition reaction is closely related to the synergistic effect of Lewis acid–base site distributions.^45,46^ The acid–base nature of the MOF material was examined using NH_3_- and CO_2_-temperature programmed desorption (TPD). The basic strength and basicity of the catalysts were classified into two groups: weak (50–176 °C) and medium to strong (176–500 °C).^47^ According to Fig. 5(a) and Table 1, the CO_2_-TPD profile of Ni–ImzAdn showed one weak peak and two strong desorption peaks at 80, 320, and 380 °C, respectively. The weak peak of CO_2_ adsorbed on Ni–ImzAdn may be related to its π–π stacking bonding between imidazole and CO_2_. In contrast, strong Lewis basic sites were probably by the binding interaction between nitrogen sites of adenine and CO_2_. The surface acidity of the Ni–ImzAdn catalyst was evaluated by the NH_3_-TPD as shown in Fig. 5(b). Surface acidity can be classified into three main groups: weak (49.85–99.85 °C), medium (149.85–199.85 °C), and strong (319.85–499.85 °C).^48^ The NH_3_-TPD profile of Ni–ImzAdn showed three strong desorption peaks at 240, 340, and 380 °C, respectively. As illustrated in Table 1, the high-temperature desorption peaks could be ascribed to hydrogen bonding interactions of amino groups in adenine and imidazole with CO_2_, and NH_3_ adsorbed on metal nodes as Brønsted acid sites. Table 2 summarizes and compares the acidity and basicity of Ni–ImzAdn with other MOF catalysts that were previously used in the synthesis of cyclic carbonates.^49–53^ Among them, it was observed that the Ni–ImzAdn catalyst exhibited the highest desorption temperature of NH_3_ and CO_2_ during temperature-programmed desorption analysis.

**Table 1:** Acid–base amounts of Ni–ImzAdn MOF catalyst

Entry	Temperature (°C)	NH_3_ desorption (mmol per g catalyst)	CO_2_ desorption (mmol per g catalyst)
1	100–270	621.0	—
2	270–355	855.3	—
3	355–400	2481.6	—
4	50–165	—	48.4
5	165–355	—	567.2
6	355–400	—	1256.1

**Table 2:** The comparative Lewis acid–base sites for Ni–ImzAdn with other MOF catalysts

Entry	CO_2_-TPD (°C)	NH_3_-TPD (°C)	MOF	Reference
1	142	122	NUC-111a^a^	49
2	142	148	IL-Au@UiO-66-NH_2_/CMC^b^	50
3	128 and 325	154, 251, and 457	MOF-508a^c^	51
4	145 and 280	270	[Zn_3_(BTC)_2_]^d^	52
5	320	115	Co-BTC^e^	53
**6**	**80, 320, and 380**	**240, 340, and 380**	**Ni–ImzAdn**	**This work**

a {(Me_2_NH_2_)_2_[Pb_5_(PTTPA)_2_(H_2_O)_3_]·2DMF·3H_2_O}_*n*_.

b (Carboxymethylcellulose (CMC); 1-aminoethyl-3-methylimidazolium bromide (IL)).

c Zn(tp)(bpy)_0.5_·(DMF)(H_2_O)_0.5_.

d Zinc-1,3,5-benzenetricarboxylate.

e [(CH_3_)_2_NH_2_][Co_3_(BTC)(HCOO)_4_(H_2_O)]·H_2_O.

Therefore, among them, Ni–ImzAdn had the most excellent catalytic performance and high synergistic effect of Lewis acid–base to facilitate the capture and conversion of CO_2_.

**Fig. 6:**
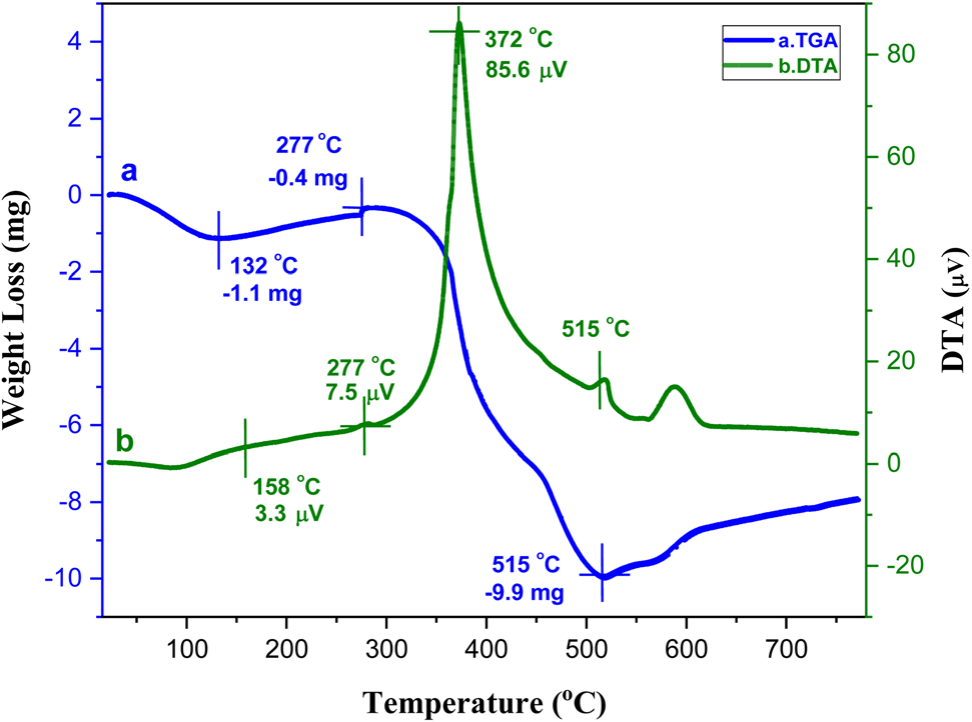
TG-DTA analysis of Ni–ImzAdn MOF.

The thermal stability of the catalyst was examined by TG-DTA (Fig. 6). The initial weight loss (5.34%) occurred below 132 °C, which is attributed to the removal of moisture from the catalyst. Subsequently, another weight loss (1.94%) at 277 °C observed due to the evaporation of guest molecules, such as solvent molecules from the cavities of the MOF. Upon further continuation of heating above 318 °C, the sample started to disintegrate until it was completely decomposed at 515 °C due to the breaking of the network, indicating the high thermal stability of the Ni–ImzAdn MOF. In DTA curve, it is observed the significant exothermic peak at 372 °C belong to the decompositions of the linkers in the framework.

### 3.2 Catalytic activity for CO_2_ cycloaddition reaction

The heterogeneous nitrogen-rich MOF catalyst was examined for CO_2_ cycloaddition reaction at ambient pressure. The catalytic performance of the Ni–ImzAdn was investigated under different operating conditions. For the present purpose, the chemical fixation of CO_2_ with SO was considered as the model reaction in Table 3. As indicated in entry 1–5 in Table 3, no yield was observed in the absence of a catalyst, Ni(NO_3_)_2_·6H_2_O, imidazole, adenine, and in the presence catalyst without co-catalyst (TBAB). According to the data presented in entry 6, the fixation proceeds to around 24% yield using a catalytic amount of TBAB (0.15 mmol) alone; however, it can be increased to 93% yield with the addition of Ni–ImzAdn as catalyst (entry 12, Table 3). This may be because of the synergistic effects between acid–base bifunctional catalyst and TBAB. Furthermore, Ni–ImzAdn with rich accessible nitrogen sites can be incorporated to effectively capture and adsorption capacity of CO_2_ through chemical fixation. The results of the chemical fixation of CO_2_ with SO at various catalyst loadings (ranging from 10.0 mg to 60.0 mg) are shown in Table 3; entries 10–14. The low amount of catalyst (10 mg) led to a lower product yield (Table 3, entry 10). The yield to cyclic carbonate was excellent (in the range 93–95%) when the amount of catalyst increased from 10 mg to 60 mg (Table 3, entry 12–14). Nevertheless, due to the lower consumption of the catalyst and the proximity of yield in entries 12–14, moderate loading of catalyst (20 mg) was chosen as the optimal catalyst loading. The effect of the temperature and time in the CO_2_ cycloaddition reaction was investigated. The optimal cyclic carbonate content was 93% at 8 h and 100 °C (Table 3, entry 12). The results show that the yield decreases with increasing temperature to 120 °C (Table 3, entry 19). Considering that the reaction takes place under ambient pressure, the ability of the catalyst to capture and conversion CO_2_ gas at 120 °C is lower than at 100 °C. The effect of various solvents such as H_2_O, ethanol, toluene, *n*-hexane, and much more was investigated. The highest yield between various solvents was achieved with DMF (73%) at a temperature of 100 °C and ambient pressure of CO_2_ for 8 h (Table 3, entry 24). The heterogeneous catalytic performance under ambient air (approximately 0.04% CO_2_) showed low yield due to the low concentration of carbon dioxide (Table 3, entry 30).

**Table 3:** The optimized reaction conditions of CO_2_ cycloaddition reaction^a^

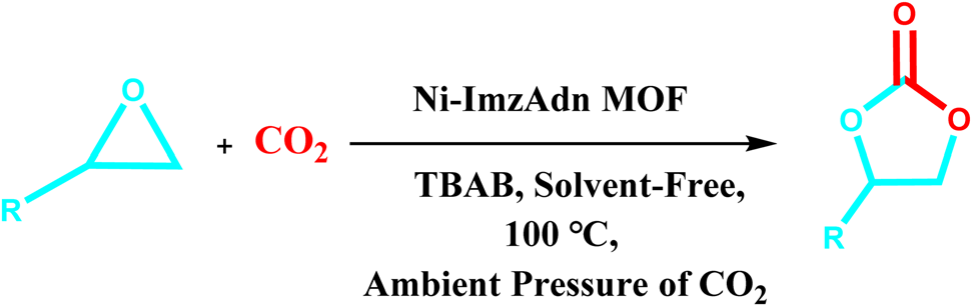							
Entry	Catalyst	Amount of catalyst (mg)	TBAB (mmol)	Temperature (°C)	Solvent	Time (h)	Yield^b^ (%)
1	No catalyst	0	0	100	Solvent-free	8	0
2	Ni(NO_3_)_2_·6H_2_O	8	0	100	Solvent-free	8	0
3	Imidazole	4	0	100	Solvent-free	8	0
4	Adenine	8	0	100	Solvent-free	8	0
5	Ni–ImzAdn	20	—	100	Solvent-free	8	0
6	—	—	0.15	100	Solvent-free	8	24
7	Ni(NO_3_)_2_·6H_2_O	8	0.15	100	Solvent-free	8	27
8	Imidazole	4	0.15	100	Solvent-free	8	38
9	Adenine	8	0.15	100	Solvent-free	8	41
10	Ni–ImzAdn	10	0.15	100	Solvent-free	8	80
11	Ni–ImzAdn	15	0.15	100	Solvent-free	8	86
**12**	**Ni–ImzAdn**	**20**	**0.15**	**100**	**Solvent-free**	**8**	**93**
13	Ni–ImzAdn	40	0.15	100	Solvent-free	8	95
14	Ni–ImzAdn	60	0.15	100	Solvent-free	8	95
15	Ni–ImzAdn	20	0.15	r.t	Solvent-free	8	7
16	Ni–ImzAdn	20	0.15	40	Solvent-free	8	11
17	Ni–ImzAdn	20	0.15	60	Solvent-free	8	33
18	Ni–ImzAdn	20	0.15	80	Solvent-free	8	54
19	Ni–ImzAdn	20	0.15	120	Solvent-free	8	86
20	Ni–ImzAdn	20	0.15	Reflux	H_2_O	8	10
21	Ni–ImzAdn	20	0.15	Reflux	Ethanol	8	30
22	Ni–ImzAdn	20	0.15	Reflux	Toluene	8	55
23	Ni–ImzAdn	20	0.15	Reflux	*n*-Hexane	8	40
24	Ni–ImzAdn	20	0.15	100	DMF	8	73
25	Ni–ImzAdn	20	0.15	100	DMSO	8	61
26	Ni–ImzAdn	20	0.15	100	Solvent-free	2	31
27	Ni–ImzAdn	20	0.15	100	Solvent-free	4	83
28	Ni–ImzAdn	20	0.15	100	Solvent-free	6	87
29	Ni–ImzAdn	20	0.15	100	Solvent-free	12	93
30	Ni–ImzAdn	20	0.15	100	Solvent-free	8	11^c^

a The CO_2_ cycloaddition reaction condition: styrene oxide (1.5 mmol), TBAB (0.15 mmol), and ambient pressure of CO_2_.

b Determined by column chromatography.

c Heterogeneous catalytic performance for fixation of CO_2_ from the ambient air (approximately 0.04 percent CO_2_ in air).

### 3.3 Catalytic activity towards different epoxides

Under the optimal reaction conditions, the coupling reaction of CO_2_ with various epoxides were investigated in the presence of Ni–ImzAdn (Table 4). It is worth mentioning that, all epoxides containing electron-donating or electron-withdrawing groups can be efficiently converted into the corresponding cyclic carbonates with good to high yield. A yield of 79% was achieved for 1,2-epoxycyclohexane, which can probably be attributed to the higher steric hindrance of the cyclohexene ring (Table 4, entry 5). ^1^H and ^13^C NMR spectra have been given in ESI.†

**Table 4:** The cycloaddition of CO_2_ with various substrates under optimized catalytic conditions: catalyst (20 mg), TBAB (0.15 mmol, 0.05 gr), epoxide (1.5 mmol), and temperature (100 °C) in ambient pressure and solvent-free condition

Entry	Reactant	Time (h)	Product	Yield^a^ (%)
1	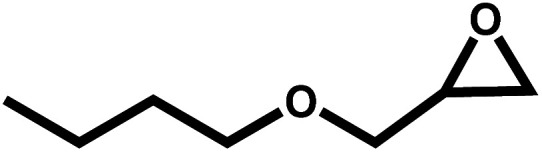	8	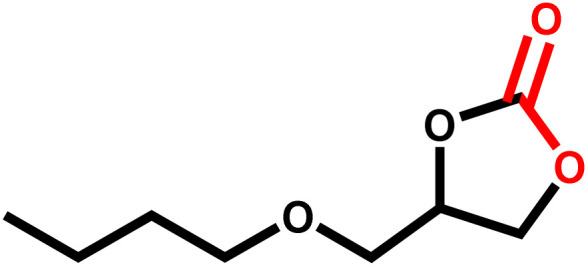	85
2	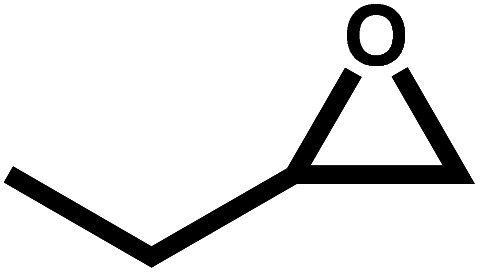	8	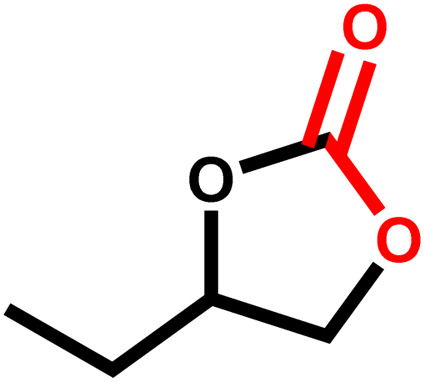	88
3	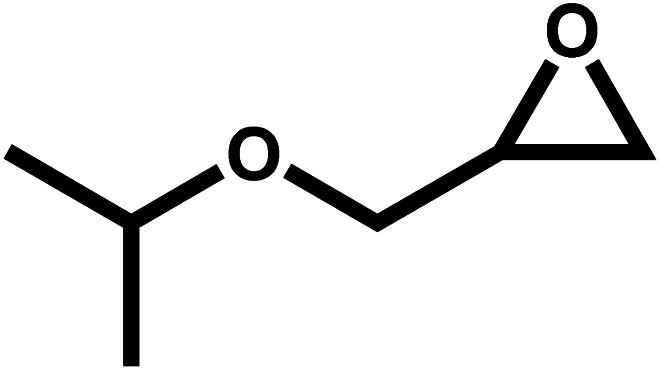	8	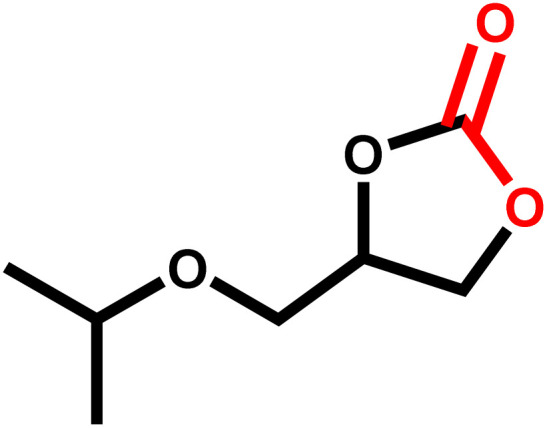	82
4	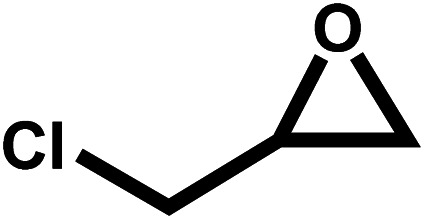	8	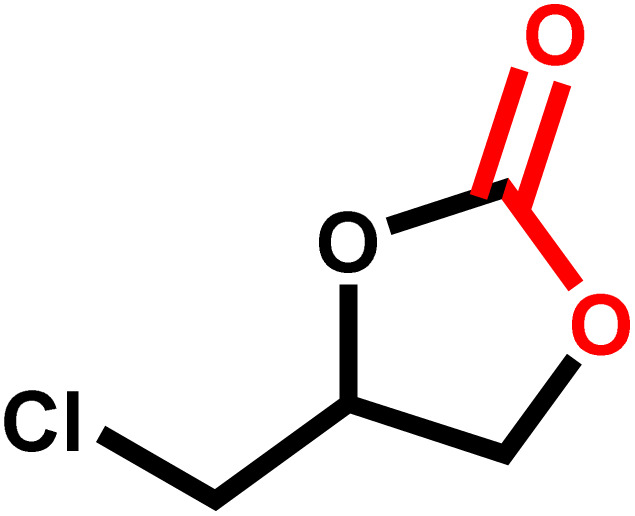	91
5	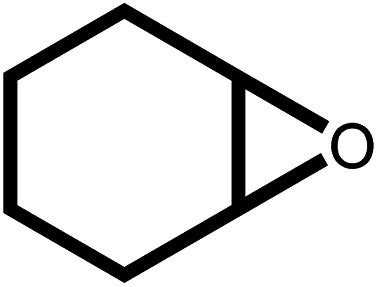	8	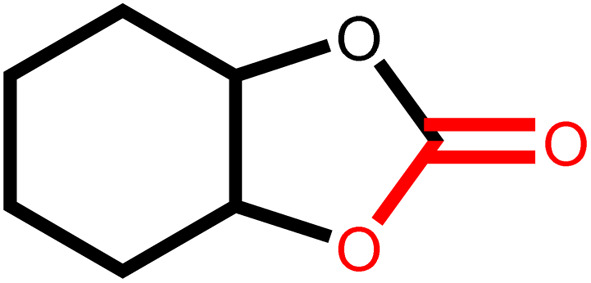	79
6	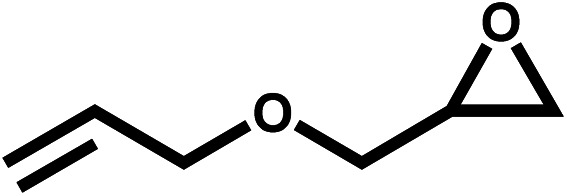	8	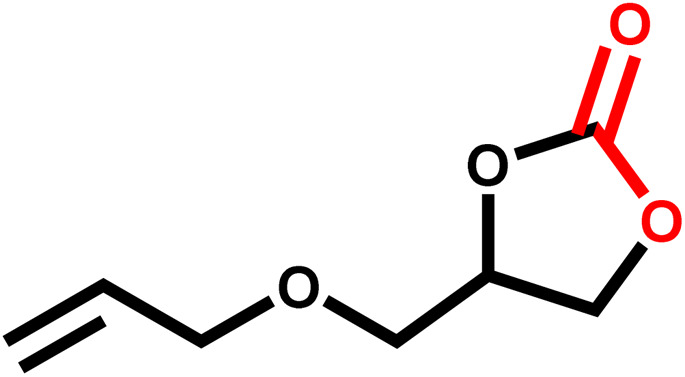	87
7	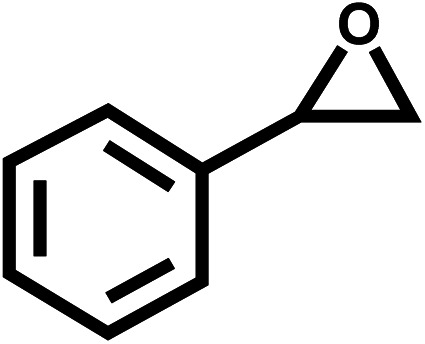	8	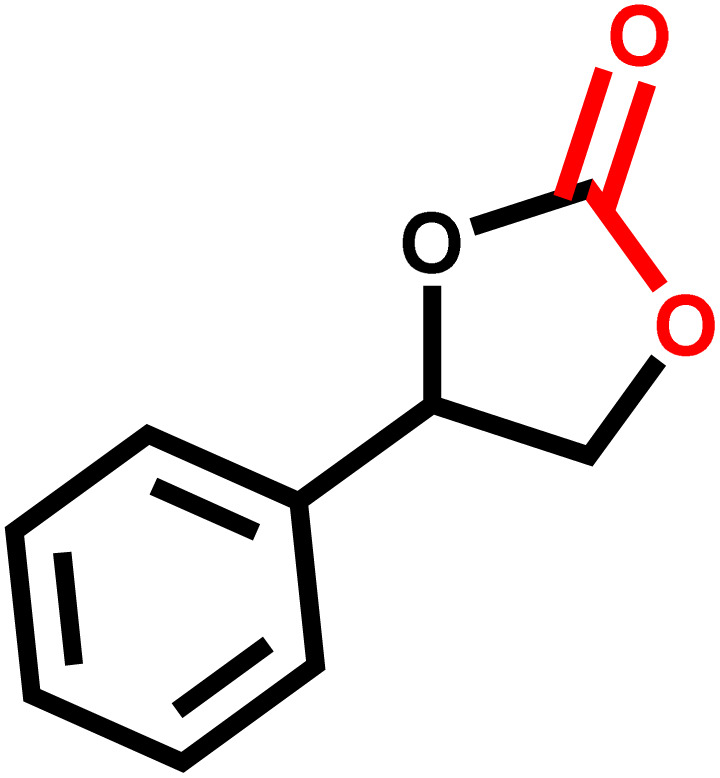	93
8	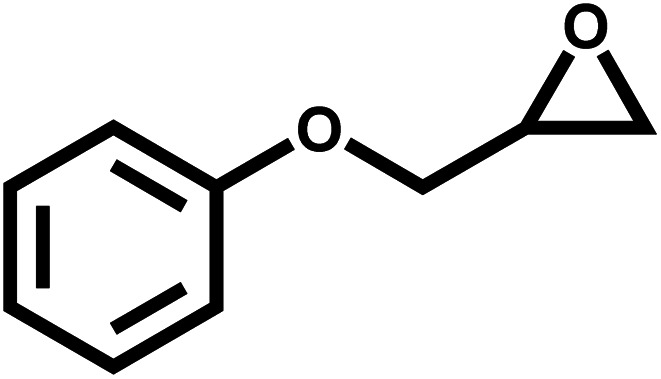	4	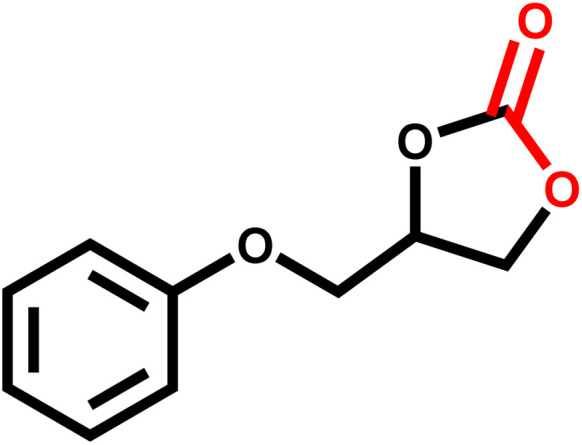	97

a The yields were obtained using column chromatography with confirmation by ^1^H and ^13^C NMR.

### 3.4 Reusability study of the MOF catalyst

The recyclability of the catalyst is a significant aspect of the research on catalytic materials. As shown in Fig. S4 (ESI†), the initial performance of the catalyst can be remained after 6 runs of cycloaddition reaction, while the yield of product decreased only slightly. FT-IR and XRD analyses were used to investigate structural changes and MOF crystallinity. Furthermore, the ICP-OES technique was used to determine nickel leaching from the MOF catalyst after six catalytic cycles. The ICP-OES technique was successfully applied to detect the metal leaching in the fresh and 6th reused Ni–ImzAdn catalyst. According to the report in Table 5, nickel decreased from 0.06731 mmol (fresh sample) to 0.06692 mmol (after the 6th run). The catalyst was reused for six reaction runs, and no significant loss of activity was observed in the cycloaddition of CO_2_ with styrene oxide. However, no significant loss of catalytic activity and low metal leaching (3.9 × 10^−4^ mmol) at the end of six reaction runs, indicating that the metal nodes were stabilized by the free nitrogen sites of the Ni–ImzAdn.

**Table 5:** The ICP-OES results of fresh and 6th run reused Ni–ImzAdn catalyst

Entry	Sample	Mass (g)	Volume (mL)	Dilution factor	Element	Instrument data (mg L^−1^)	Conversion content (mg kg^−1^)
1	Fresh catalyst	0.02	25	100	Ni	158.085	197 535
2	Catalyst (6th run)	0.02	25	100	Ni	157.177	196 400

As shown in Fig. S5 (ESI†), after six reaction cycles, no chemical change in the heterogeneous catalyst structure was observed by FT-IR spectroscopy, that frequencies and intensities of all absorption bands are well-preserved. Additionally, as shown by the XRD patterns in Fig. S6 (ESI†), the XRD analysis of the 6th run reused Ni–ImzAdn catalyst shows the same positions of the XRD pattern in the fresh catalyst and a very slight decrease in intensity of the Ni–O peaks, which can be due to the trace loosening the metal nodes of MOF catalyst. Table 6, shows the comparative performance of Ni–ImzAdn MOF catalyst with other MOF-based catalysts reported in the literature for the conversion of styrene oxide to cyclic carbonate using CO_2_ under solvent-free conditions.^54–58^ As shown in Table 6, entry 2–6, for all catalysts much higher CO_2_ pressures are usually needed to achieve high conversion (from 8 to 20 bar in one case) at the closest temperature conditions. For example, similar conditions were employed by Verpoort and coworkers, with a ZIF-67 MOF,^54^ and Park and coworkers,^57^ with Cu-MOF in both the catalytic system, high pressure is required for CO_2_ fixation. On the opposite side, higher cyclic carbonate conversion (95.0%) was found to be at the ambient pressure by Ni–ImzAdn MOF catalyst. These results showed that the Ni–ImzAdn MOF has a high affinity for absorbing CO_2_ to carry out the fixation process.

**Table 6:** The comparative catalytic activity of Ni–ImzAdn MOF catalyst with reported MOFs known for cycloaddition of CO_2_ with SO, using TBAB as co-catalyst, at the closest temperature conditions

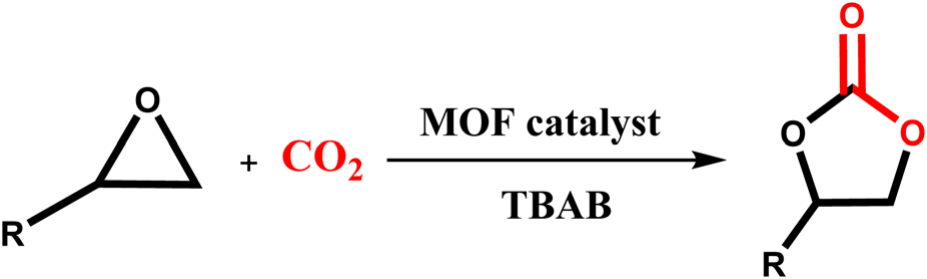						
Entry	MOF catalyst	Time (h)	Temperature (°C)	Pressure (bar)	Conversion (%)	Ref.
**1**	**Ni–ImzAdn**	**8**	**100**	**Ambient**	**95.0**	**This work**
2	ZIF-67	15	100	10	92.0	54
3	MIL-68(In)-NH_2_	8	150	8	74.0	55
4	[Cu_2_L(H_2_O)_2_]·4H_2_O·2DMF	6	100	10	64.1	56
5	{Cu(Hip)_2_(Bpy)}_*n*_(CHB)	6	120	12	69.8	57
6	Gea-MOF-1	6	120	20	85.1	58

### 3.5 Possible reaction mechanism

**Fig. 7:**
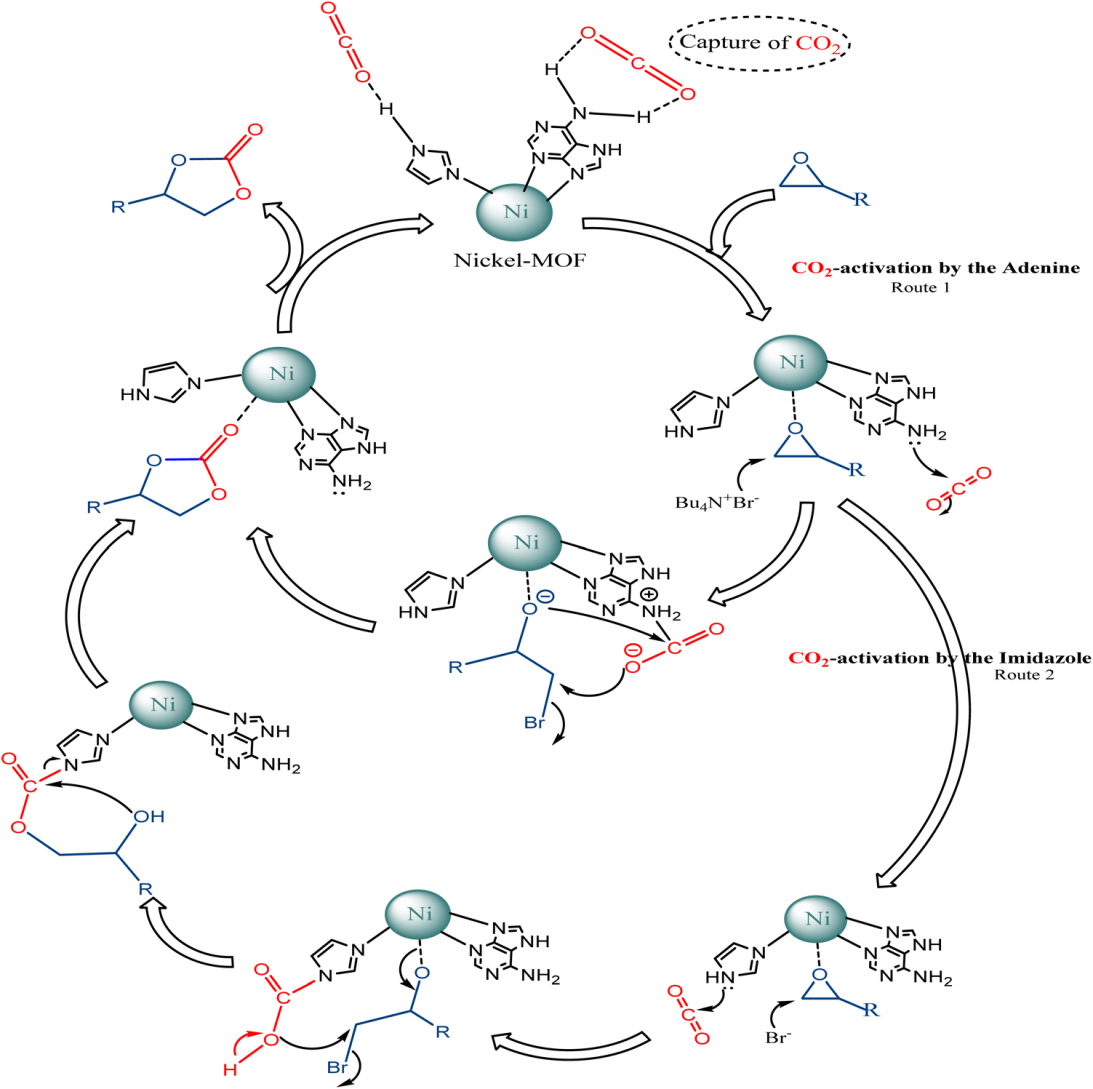
The proposed mechanism for the chemical fixation of CO_2_ using nickel-MOF/TBAB system.

According to previous literature,^59–61^ a plausible reaction mechanism of the CO_2_/epoxide coupling by Ni–ImzAdn is shown in Fig. 7. As expected, the microporous Ni–ImzAdn MOF with polar uncoordinated N-rich sites have good CO_2_ uptake capacity due to the presence of adenine and imidazole inside the channels. On route number 1, the activation of CO_2_ occurs through a nucleophilic attack by adenine on the carbon atom of CO_2_ and the metal nodes provide acid sites for epoxide activation to promote ring-opening of the epoxide. In the second step of route 1, ring-opening of the epoxide takes place by a nucleophilic attack of the (Br^−^) anion from TBAB on the least-hindered carbon atom of Ni-epoxide to form metal-coordinated bromo-alkoxide. For the next step, the resulting alkoxide-ion reacts with the polarized CO_2_ molecule, which can cause the intramolecular cyclization to give the corresponding carbonate. On route number 2, the activation of CO_2_ occurs through a nucleophilic attack by –NH groups in the axial imidazole rings on the carbon atom of CO_2_ and the metal nodes provide acid sites for epoxide activation to promote ring-opening of the epoxide. Ring-opening of the epoxide takes place by a nucleophilic attack of the (Br^−^) anion from TBAB on the least-hindered carbon atom of Ni-epoxide to form metal-coordinated bromo-alkoxide. Finally, alkoxide-ion reacts with carbamic acid, then intramolecular cyclization occurs to give carbonate.^24^

## 4. Conclusion

In summary, Ni-based MOF, having strong Lewis acid-base dual-functional sites, high porosity, and amino-functional channels for high capture of CO_2_ gas molecules, was investigated towards catalytic cycloaddition of CO_2_ to epoxides to afford versatile and useful cyclic carbonate compounds under solvent-free condition. Also, due to the presence of N-containing ligands (imidazole and adenine) and open Ni(ii) metal sites as high-density basic and acidic centers respectively, in comparison with many conventional MOFs that perform the CO_2_ fixation at high pressure, this MOF carried out the reaction at ambient pressure. Moreover, the catalyst exhibited high thermal and structural stability, along with excellent reusability, showing no significant decline in catalytic activity even after six cycles. These findings highlight the potential of this MOF as an efficient and durable heterogeneous catalyst.

## Data availability

The data supporting this article have been included as part of the ESI.†

## Author contributions

Reza Erfani-Ghorbani: writing – original draft, methodology, investigation, formal analysis, data curation. Hossein Eshghi: supervision, conceptualization, writing – review & editing. Ali Shiri: supervision, writing – review & editing.

## Conflicts of interest

The authors declare that they have no known competing financial interests or personal relationships that could have appeared to influence the work reported in this paper.

## Supplementary Material

RA-015-D4RA08614G-s001

## References

[cit1] Kim C., Yoo C.-J., Oh H.-S., Min B. K., Lee U. (2022). Review of carbon dioxide utilization technologies and their potential for industrial application. J. CO_2_ Util..

[cit2] Zhao L., Hu H.-Y., Wu An-G., Terent’ev A. O., He L.-N., Li H.-R. (2024). CO_2_ capture and *in situ* conversion to organic molecules. J. CO_2_ Util..

[cit3] Fiorani G., Perosa A., Selva M. (2023). Sustainable valorisation of renewables through dialkyl carbonates and isopropenyl esters. Green Chem..

[cit4] Song Q.-W., Ma R., Liu P., Zhang K., He L.-N. (2023). Recent progress in CO_2_ conversion into organic chemicals by molecular catalysis. Green Chem..

[cit5] Aytar E., Yasar E., Kilic A. (2024). The efficient and reusable imidazolium-organoboron catalysts for green CO_2_ insertion reactions in solvent-free under atmospheric and high-pressure conditions. Fuel.

[cit6] Rehman A., Saleem F., Javed F., Ikhlaq A., Ahmad S. W., Harvey A. (2021). Recent advances in the synthesis of cyclic carbonates *via* CO_2_ cycloaddition to epoxides. J. Environ. Chem. Eng..

[cit7] Yan T., Liu H., Zeng Z. X., Pan W. G. (2023). Recent progress of catalysts for synthesis of cyclic carbonates from CO_2_ and epoxides. J. CO_2_ Util..

[cit8] Ravi S., Kim J., Choi Y., Han H. H., Wu S., Xiao R., Bae Y.-S. (2023). Metal-free amine-anchored triazine-based covalent organic polymers for selective CO_2_ adsorption and conversion to cyclic carbonates under mild conditions. ACS Sustainable Chem. Eng..

[cit9] Saha E., Jungi H., Dabas S., Mathew A., Kuniyil R., Subramanian S., Mitra J. (2023). Amine-rich nickel(ii)-xerogel as a highly active bifunctional metallo-organo catalyst for aqueous knoevenagel condensation and solvent-free CO_2_ cycloaddition. Inorg. Chem..

[cit10] Pourhassan F., Khalifeh R., Eshghi H. (2021). Well dispersed gold nanoparticles into the multi amine functionalized SBA-15 for green chemical fixation of carbon dioxide to cyclic carbonates under solvent free conditions. Fuel.

[cit11] Usman M., Rehman A., Saleem F., Abbas A., Eze V. C., Harvey A. (2023). Synthesis of cyclic carbonates from CO_2_ cycloaddition to bio-based epoxides and glycerol: an overview of recent development. RSC Adv..

[cit12] Chuang J.-Y., Liu K.-T., Lin M. M., Yu W.-Y., Jeng Ru-J., Leung M.-K. (2023). Two novel types of heterogeneous catalysts apply to synthesize cyclic carbonates through CO_2_ fixation on epoxides under mild condition. J. CO2 Util..

[cit13] Tyagi P., Singh D., Malik N., Kumar S., Singh Malik R. (2023). Metal catalyst for CO_2_ capture and conversion into cyclic carbonate: progress and challenges. Mater. Today.

[cit14] Wang K., Li H., Lin Y., Luo Yu-Z., Yao Zi-J. (2024). Heterogeneous catalytic conversion of carbon dioxide and epoxides to cyclic carbonates. Surf. Interfaces.

[cit15] Liu Y., Li S., Chen Y., Li M., Chen Z., Hu T., Shi L., Pudukudy M., Shan S., Zhi Y. (2023). Urea/amide-functionalized melamine-based organic polymers as efficient heterogeneous catalysts for CO_2_ cycloaddition. Chem. Eng. J..

[cit16] Wan Y.-L., Wang L., Wen L. (2022). Amide-functionalized organic cationic polymers toward enhanced catalytic performance for conversion
of CO_2_ into cyclic carbonates. J. CO_2_ Util..

[cit17] Kim D., Kyungsu Na. (2018). Organic–inorganic multifunctional hybrid catalyst giving catalytic synergies in cooperative coupling between CO_2_ and propylene oxide to propylene carbonate. J. CO_2_ Util..

[cit18] Wang B., Wang L., Lin J., Xia C., Sun W. (2023). Multifunctional Zn-N4 catalysts for the coupling of CO_2_ with epoxides into cyclic carbonates. ACS Catal..

[cit19] Obi C. C., Nwabanne J. T., Kanuria Igbokwe P., Idumah C. I., Okpechi V. U., Oyeoka H. C. (2024). Novel advances in synthesis and catalytic applications of metal–organic frameworks-based nanocatalysts for CO_2_ capture and transformation. J. Environ. Chem. Eng..

[cit20] Gulati S., Vijayan S., Kumar S., Harikumar B., Trivedi M., Varma R. S. (2023). Recent advances in the application of metal–organic frameworks (MOFs)-based nanocatalysts for direct conversion of carbon dioxide (CO_2_) to value-added chemicals. Coord. Chem. Rev..

[cit21] Hu L., Xu W., Jiang Q., Ji R., Yan Z., Wu G. (2024). Recent progress on CO_2_ cycloaddition with epoxide catalyzed by ZIFs and ZIFs-based materials. J. CO_2_ Util..

[cit22] Khattak Z. A. K., Ahmad N., Younus H. A., Ullah H., Yu B., Munawar K. S., Ashfaq M. (2024). *et al.*, Ambient conversion of CO_2_ and epoxides to cyclic carbonates using 3D amide-functionalized MOFs. Catal. Sci. Technol..

[cit23] Eskemech A., Chand H., Karmakar A., Krishnan V., Koner R. R. (2024). Zn-MOF as a single catalyst with dual lewis acidic and basic reaction sites for CO_2_ fixation. Inorg. Chem..

[cit24] Kuruppathparambil R. R., Robert T. M., Pillai R. S., Pillai S. K. B., Shankaranarayanan S. K. K., Kim D., Mathew D. (2022). Nitrogen-rich dual linker MOF catalyst for room temperature fixation of CO_2_*via* cyclic carbonate synthesis: DFT assisted mechanistic study. J. CO_2_ Util..

[cit25] Hu Y., Abazari R., Sanati S., Nadafan M., Carpenter-Warren C. L., Slawin A. M. Z., Zhou Y., Kirillov A. M. (2023). A dual-purpose Ce(iii)–organic framework with amine groups and open metal sites: third-order nonlinear optical activity and catalytic CO_2_ fixation. ACS Appl. Mater. Interfaces.

[cit26] Yang X.-L., Yan Y.-T., Wang W.-J., Ze-Ze H., Zhang W.-Y., Huang W., Wang Y.-Y. (2021). A 2-fold interpenetrated nitrogen-rich metal–organic framework: dye adsorption and CO_2_ capture and conversion. Inorg. Chem..

[cit27] Nguyen Q. T., Na J., Lee Y.-R., Baek K.-Y. (2024). Boosting catalytic performance for CO_2_ cycloaddition under mild condition *via* amine grafting on MIL-101-SO_3_H catalyst. J. Environ. Chem. Eng..

[cit28] Saghian M., Dehghanpour S., Sharbatdaran M. (2020). Amine-functionalized frameworks as highly actives catalysts for chemical fixation of CO_2_ under solvent and co-catalyst free conditions. J. CO_2_ Util..

[cit29] Zulys A., Yulia F., Muhadzib N., Nasruddin N. (2020). Biological metal–organic frameworks (Bio-MOFs) for CO_2_ capture. Ind. Eng. Chem. Res..

[cit30] Mohamed H., Syahirah N., Mohamed K. A., Hussin F., Ti Gew L. (2022). A systematic review of amino acid-based adsorbents for CO_2_ capture. Energies.

[cit31] Erzina M., Guselnikova O., Elashnikov R., Trelin A., Zabelin D., Postnikov P., Siegel J. (2023). *et al.*, BioMOF coupled with plasmonic CuNPs for sustainable CO_2_ fixation in cyclic carbonates at ambient conditions. J. CO_2_ Util..

[cit32] Gupta R. K., Riaz M., Ashafaq M., Gao Z.-Y., Varma R. S., Li D.-C., Cui P., Tung C.-H., Sun D. (2022). Adenine-incorporated metal–organic frameworks. Coord. Chem. Rev..

[cit33] Pettinari C., Tombesi A. (2020). Metal–organic frameworks for carbon dioxide capture. MRS Energy Sustainability.

[cit34] Shen F., Wu J., Chen G., Wei Z., Wang J., Lin Z., Chai K. (2024). Efficient capture of CO_2_ from flue gas and biogas by moisture-stable adenine-based ultramicroporous metal–organic framework. J. Environ. Chem. Eng..

[cit35] Rachuri Y., Kurisingal J. F., Kumar Chitumalla R., Vuppala S., Gu Y., Jang J., Choe Y., Suresh E., Park D.-W. (2019). Adenine-based Zn(ii)/Cd(ii) metal–organic frameworks as efficient heterogeneous catalysts for facile CO_2_ fixation into cyclic carbonates: a DFT-supported study of the reaction mechanism. Inorg. Chem..

[cit36] Lee H. M., Youn Il S., Saleh M., Lee J. W., Kim K. S. (2015). Interactions of CO_2_ with various functional molecules. Phys. Chem. Chem. Phys..

[cit37] Agarwal R. A., Gupta N. K. (2017). CO_2_ sorption behavior of imidazole, benzimidazole and benzoic acid-based coordination polymers. Coord. Chem. Rev..

[cit38] Vanaraj R., Vinodh R., Periyasamy T., Madhappan S., Mohan Babu C., Asrafali S. P., Haldhar R. (2022). *et al.*, Capacitance enhancement of metal–organic framework (MOF) materials by their morphology and structural formation. Energy Fuels.

[cit39] Zeraati M., Alizadeh V., Kazemzadeh P., Safinejad M., Kazemian H., Sargazi G. (2022). A new nickel metal organic framework (Ni-MOF) porous nanostructure as a potential novel electrochemical sensor for detecting glucose. J. Porous Mater..

[cit40] Spokoyny A. M., Kim D., Sumrein A., Mirkin C. A. (2009). Infinite coordination polymer nano-and microparticle structures. Chem. Soc. Rev..

[cit41] Shaw E. V., Chester A. M., Robertson G. P., Castillo-Blas C., Bennett T. D. (2024). Synthetic and analytical considerations for the preparation of amorphous metal–organic frameworks. Chem. Sci..

[cit42] Das S., Heasman P., Ben T., Qiu S. (2017). Porous organic materials: strategic design and structure–function correlation. Chem. Rev..

[cit43] Wen H.-M., Liao C., Li L., Ali A., Alothman Z., Krishna R., Wu H., Zhou W., Hu J., Chen B. (2019). A metal–organic framework with suitable pore size and dual functionalities for highly efficient post-combustion CO_2_ capture. J. Mater. Chem. A.

[cit44] Essalhi M., Mohan M., Dissem N., Ferhi N., Abidi A., Maris T., Duong A. (2023). Two different pore architectures of cyamelurate-based metal–organic frameworks for highly selective CO_2_ capture under ambient conditions. Inorg. Chem. Front..

[cit45] Gu J., Sun X., Liu X., Yang Y., Shan H., Liu Y. (2020). Highly efficient synergistic CO_2_ conversion with epoxide using copper polyhedron-based MOFs with Lewis acid and base sites. Inorg. Chem. Front..

[cit46] Natarajan S., Manna K. (2023). Bifunctional MOFs in heterogeneous catalysis. ACS Org. Inorg. Au.

[cit47] Jiang G., Zhang L., Zhao Z., Zhou X., Duan A., Xu C., Gao J. (2008). Highly effective P-modified HZSM-5 catalyst for the cracking of C4 alkanes to produce light olefins. Appl. Catal., A.

[cit48] Topsøe N.-Y., Pedersen K., Derouane E. G. (1981). Infrared and temperature-programmed desorption study of the acidic properties of ZSM-5-type zeolites. J. Catal..

[cit49] Zhao B., Li C., Hu T., Gao Y., Fan L., Zhang X. (2024). Robust {Pb10}-cluster-based metal–organic framework for capturing and converting CO_2_ into cyclic carbonates under mild conditions. Inorg. Chem..

[cit50] Zhao X., Chang G., Xu H., Yao Y., Dong D., Yang S., Tian G., Yang X. (2024). A hierarchical metal–organic framework composite aerogel catalyst containing integrated acid, base, and metal sites for the one-pot catalytic synthesis of cyclic carbonates. ACS Appl. Mater. Interfaces.

[cit51] Song X., Wu Y., Pan D., Zhang J., Xu S., Gao L., Wei R., Zhang J., Xiao G. (2018). Dual-linker metal–organic frameworks as efficient carbon dioxide conversion catalysts. Appl. Catal., A.

[cit52] Feng C., Cao X., Zhang L., Guo C., Akram N., Wang J. (2018). Zn 1, 3, 5-benzenetricarboxylate as an efficient catalyst for the synthesis of cyclic carbonates from CO_2_. RSC Adv..

[cit53] Wu Y., Song X., Xu S., Zhang J., Zhu Y., Gao L., Xiao G. (2019). 2-Methylimidazole modified Co-BTC MOF as an efficient catalyst for chemical fixation of carbon dioxide. Catal. Lett..

[cit54] Mousavi B., Chaemchuen S., Moosavi B., Luo Z., Gholampour N., Verpoort F. (2016). Zeolitic imidazole framework-67 as an efficient heterogeneous catalyst for the conversion of CO_2_ to cyclic carbonates. New J. Chem..

[cit55] Lescouet T., Chizallet C., Farrusseng D. (2012). The origin of the activity of amine-functionalized metal–organic frameworks in the catalytic synthesis of cyclic carbonates from epoxide and CO_2_. ChemCatChem.

[cit56] Gao C.-Y., Tian H.-R., Ai J., Lei-Jiao L., Song D., Lan Y.-Q., Sun Z.-M. (2016). A microporous Cu-MOF with optimized open metal sites and pore spaces for high gas storage and active chemical fixation of CO_2_. Chem. Commun..

[cit57] Kathalikkattil A. C., Kim D.-W., Tharun J., Soek H.-G., Roshan R., Park D.-W. (2014). Aqueous-microwave synthesized carboxyl functional molecular ribbon coordination framework catalyst for the synthesis of cyclic carbonates from epoxides and CO_2_. Green Chem..

[cit58] Beyzavi M. H., Stephenson C. J., Liu Y., Karagiaridi O., Hupp J. T., Farha O. K. (2015). Metal–organic framework-based catalysts: chemical fixation of CO_2_ with epoxides leading to cyclic organic carbonates. Front. Energy Res..

[cit59] Senthilkumar S., Maru M. S., Somani R. S., Bajaj H. C., Neogi S. (2018). Unprecedented NH_2_-MIL-101 (Al)/*n*-Bu_4_NBr system as solvent-free heterogeneous catalyst for efficient synthesis of cyclic carbonates *via* CO_2_ cycloaddition. Dalton Trans..

[cit60] Das R., Singh Dhankhar S., Nagaraja C. M. (2020). Construction of a bifunctional Zn(ii)–organic framework containing a basic
amine functionality for selective capture and room temperature fixation of CO_2_. Inorg. Chem. Front..

[cit61] Kurisingal J. F., Rachuri Y., Gu Y., Kumar Chitumalla R., Vuppala S., Jang J., Kumar Bisht K., Suresh E., Park D.-W. (2020). Facile green synthesis of new copper-based metal–organic frameworks: experimental and theoretical study of the CO_2_ fixation reaction. ACS Sustainable Chem. Eng..

