# Supplementary material for: Nitrogen-rich metal–organic framework of nickel(ii) as a highly efficient and reusable catalyst for the synthesis of cyclic carbonates at ambient pressure of CO2
Source: RSC Adv. 2025 Mar 6;15(10):7236–47. doi: 10.1039/d4ra08614g (PMC11883551; doi:10.1039/d4ra08614g)

Supplementary Information (SI) for RSC Advances.
This journal is © The Royal Society of Chemistry 2025

Supporting Information

Nitrogen-rich metal-organic framework of nickel (II) as a highly efficient and reusable catalyst for the synthesis of cyclic carbonates at ambient pressure of CO_2

Reza Erfani-Ghorbani, Hossein Eshghi *, Ali Shiri

Department of Chemistry, Faculty of Science, Ferdowsi University of Mashhad, Mashhad 9177948974, Iran

Corresponding author: heshghi@um.ac.ir

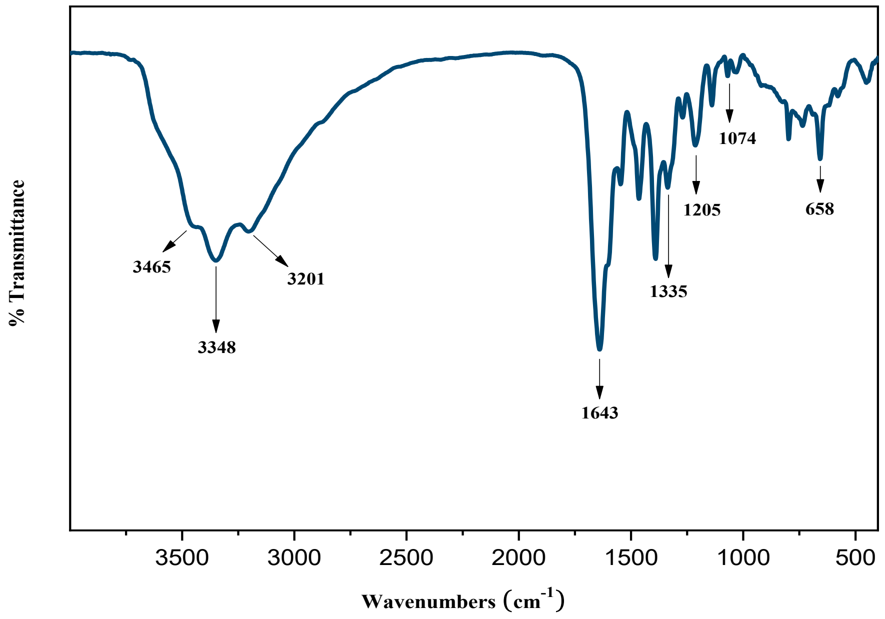

Fig. S1. The FT-IR spectrum of the Ni-ImzAdn.

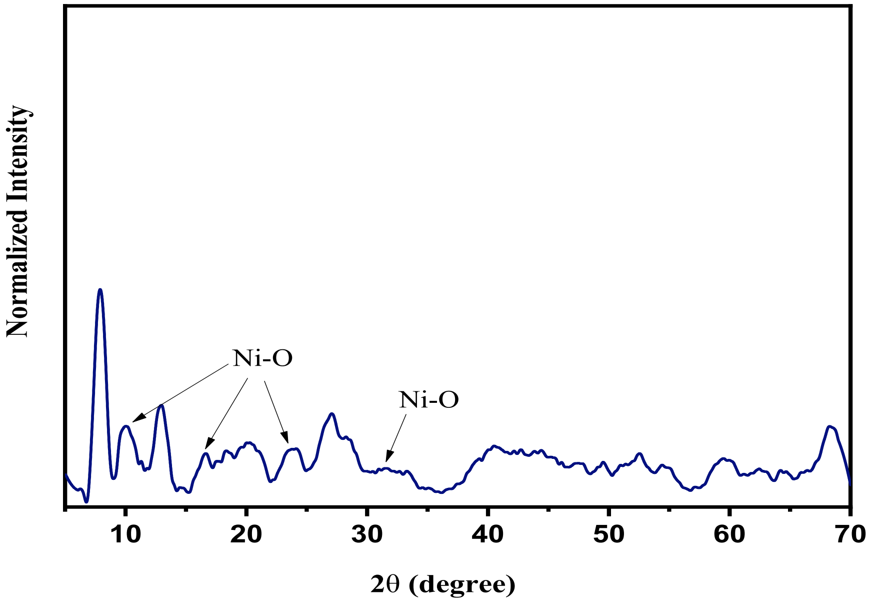

Fig. S2. The XRD patterns of Ni-ImzAdn MOF.

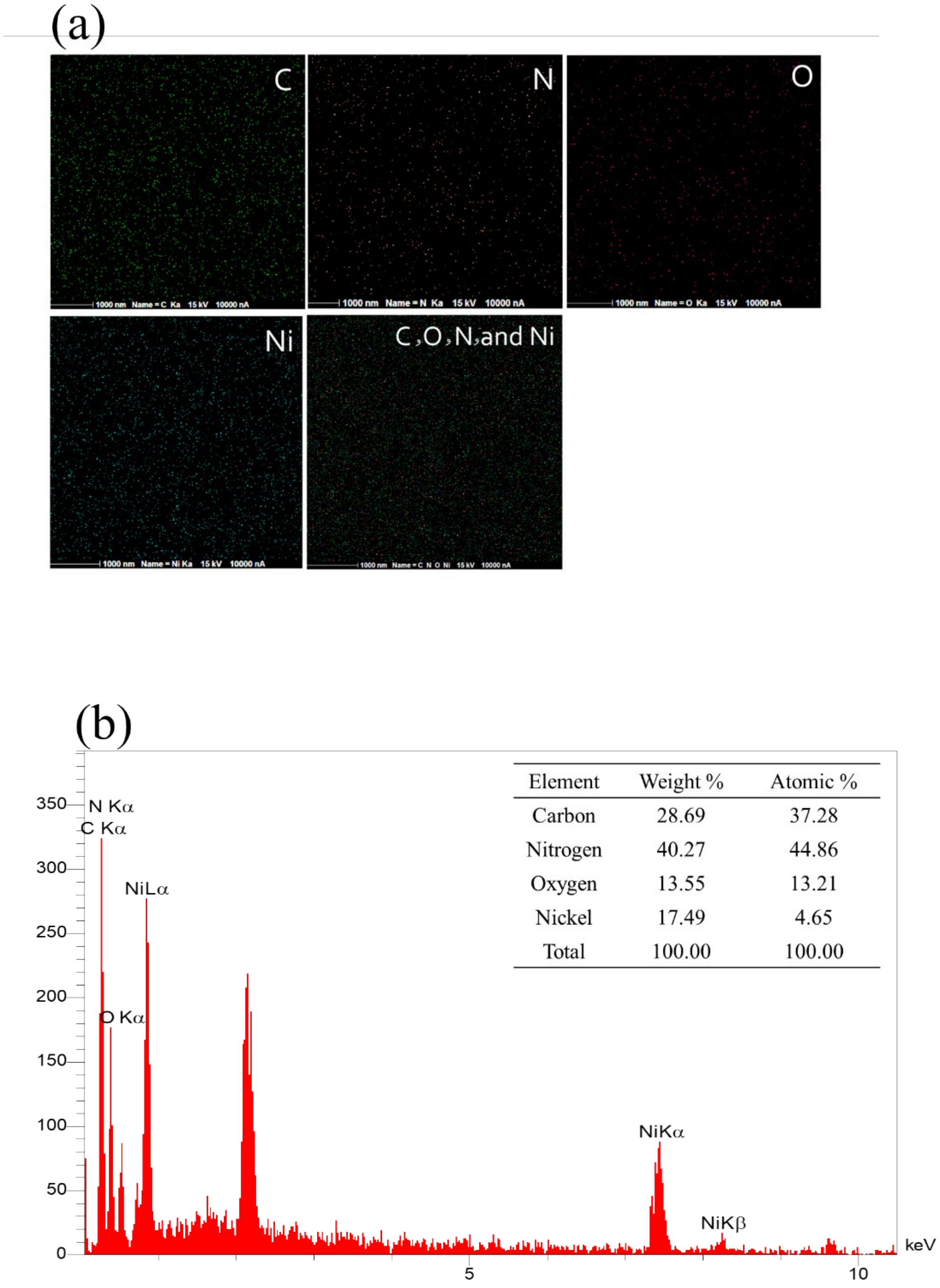

Element	Weight %	Atomic %
Carbon	28.69	37.28
Nitrogen	40.27	44.86
Oxygen	13.55	13.21
Nickel	17.49	4.65
Total	100.00	100.00

Fig. S3. Element mapping (a), and SEM-EDX analysis results (b) of Ni-ImzAdn MOF.

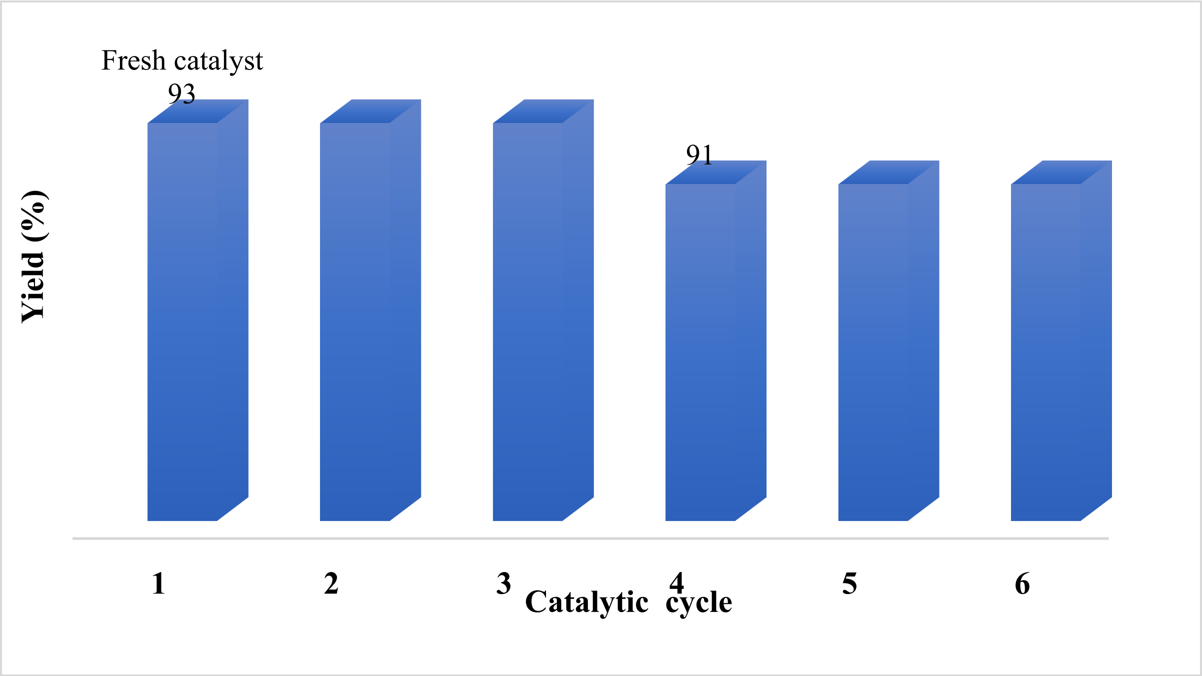

Fig. S4. Recyclability test of Ni-ImzAdn MOF catalyst for six consecutive cycles.

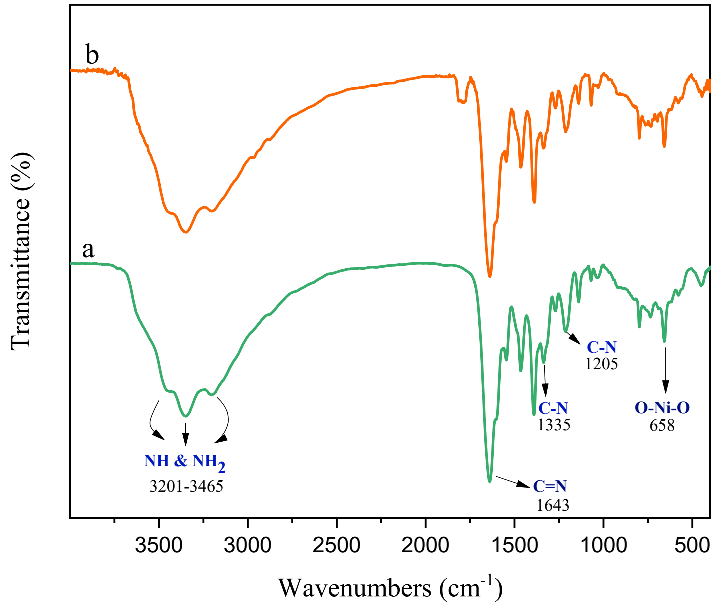

Fig. S5. FT-IR spectra fresh Ni-ImzAdn MOF catalyst (a), 6th reused Ni-ImzAdn MOF catalyst (b).

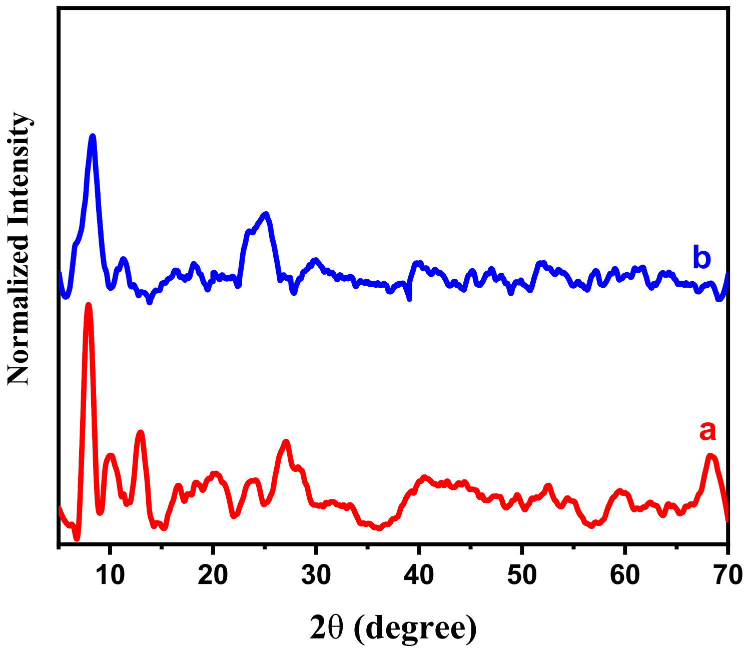

Fig. S6. XRD patterns fresh Ni-ImzAdn MOF catalyst (a), 6th reused Ni-ImzAdn MOF catalyst (b).

^{1}H NMR and ^{13}C NMR Details of Cyclic Carbonates

1. ^{1}H NMR spectrum of 4-(butoxymethyl)-1,3-dioxolan-2-one

^{1}H NMR (300 MHz, $CDCl_3$) δ 0.91 (t, J = 7.3 Hz, 3H), 1.28 – 1.43 (m, 2H), 1.55 (p, J = 6.7 Hz, 2H), 3.51 (t, J = 6.5 Hz, 2H), 3.64 (qd, J = 11.1, 3.3 Hz, 2H), 4.35 – 4.44 (m, 1H), 4.50 (t, J = 8.0 Hz, 1H), 4.76 – 4.89 (m, 1H).

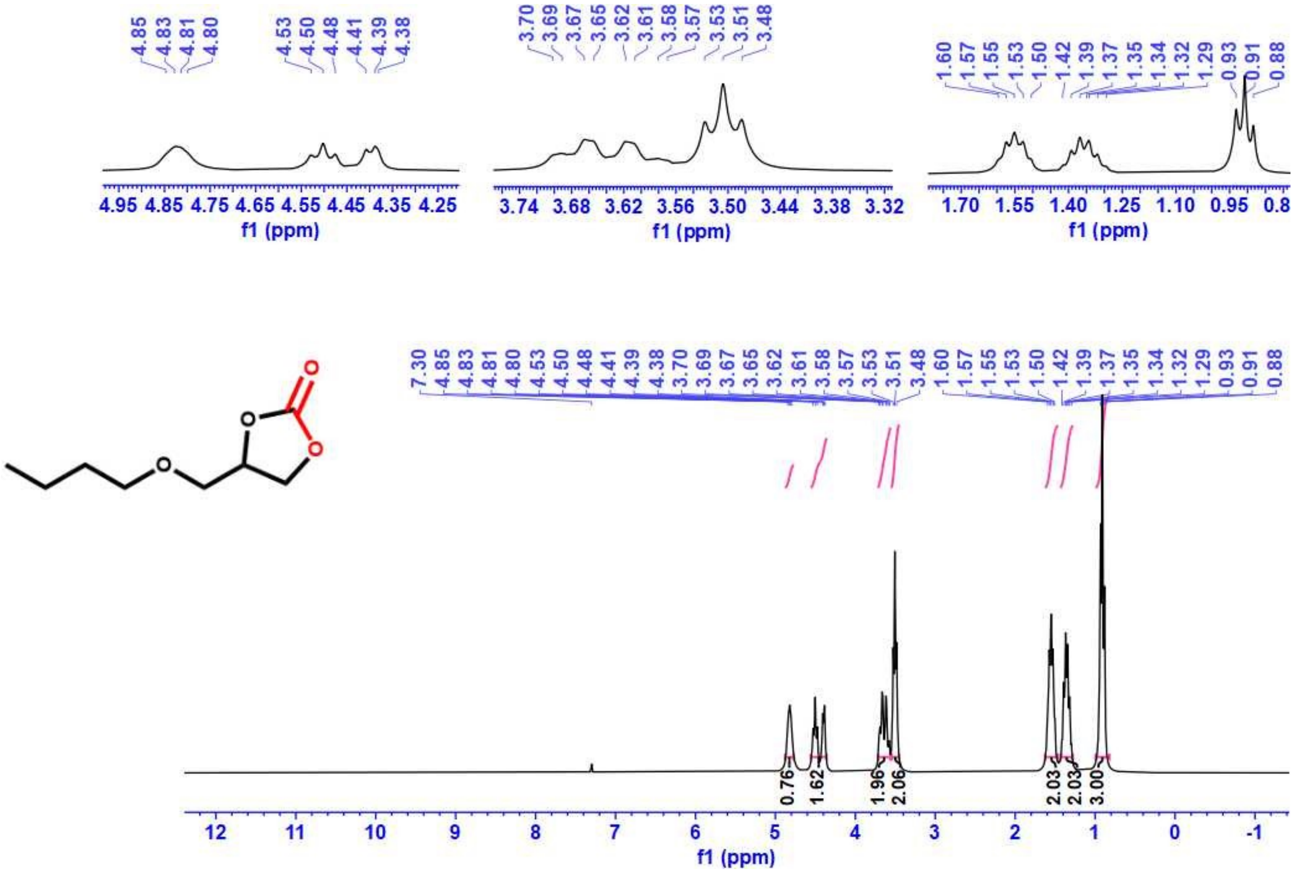

2. ^{13}C NMR spectrum of 4-(butoxymethyl)-1,3-dioxolan-2-one

^{13}C NMR (75 MHz, $CDCl_3$) δ 13.67, 18.98, 31.35, 66.22, 69.50, 71.69, 75.14, 154.99.

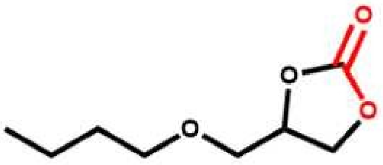

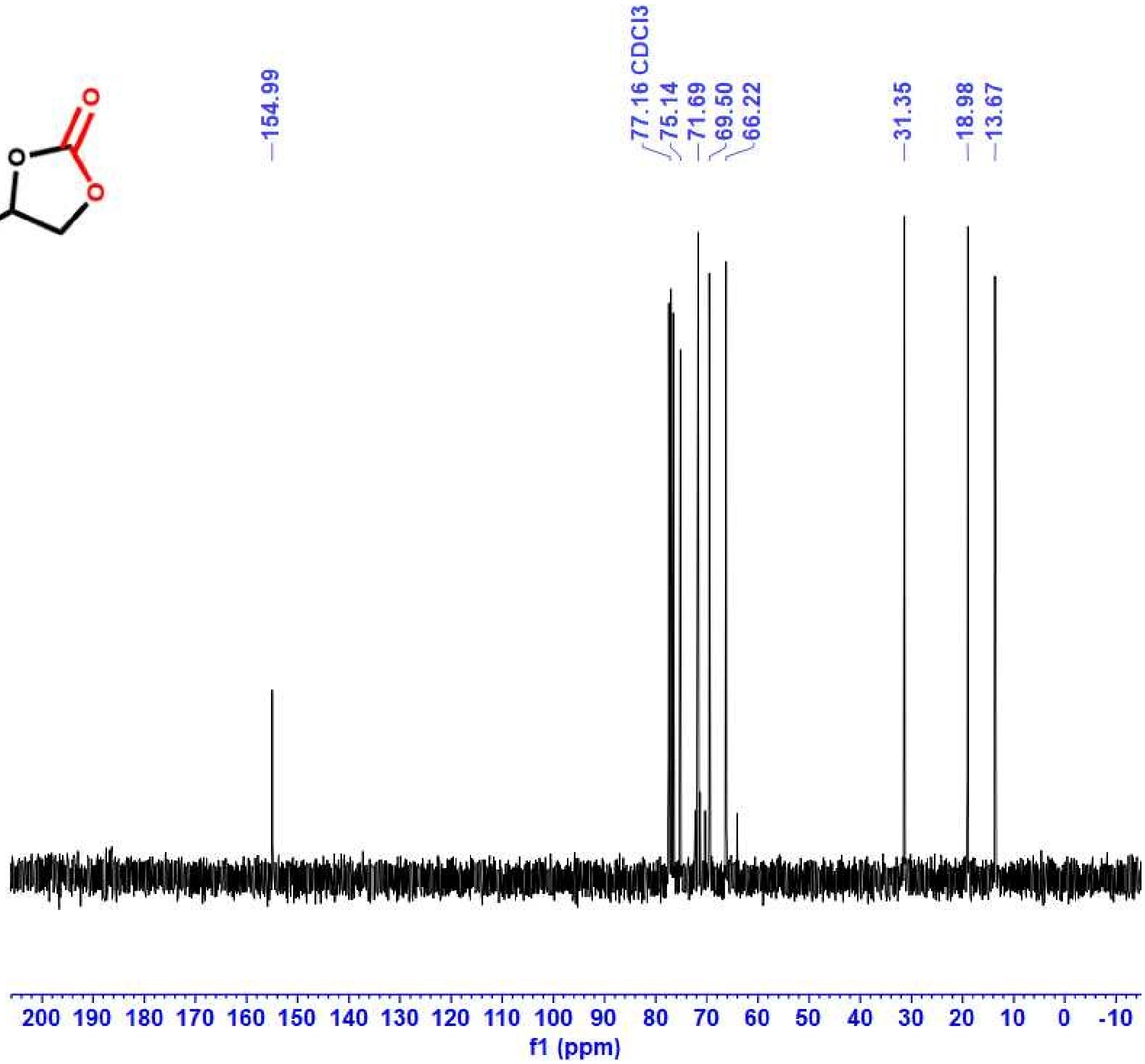

3. ^{1}H NMR spectrum of 4-phenyl-1,3-dioxolan-2-one

^{1}H NMR (300 MHz, $CDCl_3$) δ 4.28 (dd, *J* = 8.6, 7.8 Hz, 1H), 4.73 (t, *J* = 8.4 Hz, 1H), 5.61 (t, *J* = 8.0 Hz, 1H), 7.30 (m, 2H), 7.37 (dt, *J* = 4.7, 2.7 Hz, 3H).

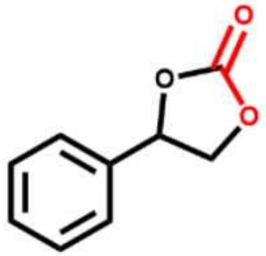

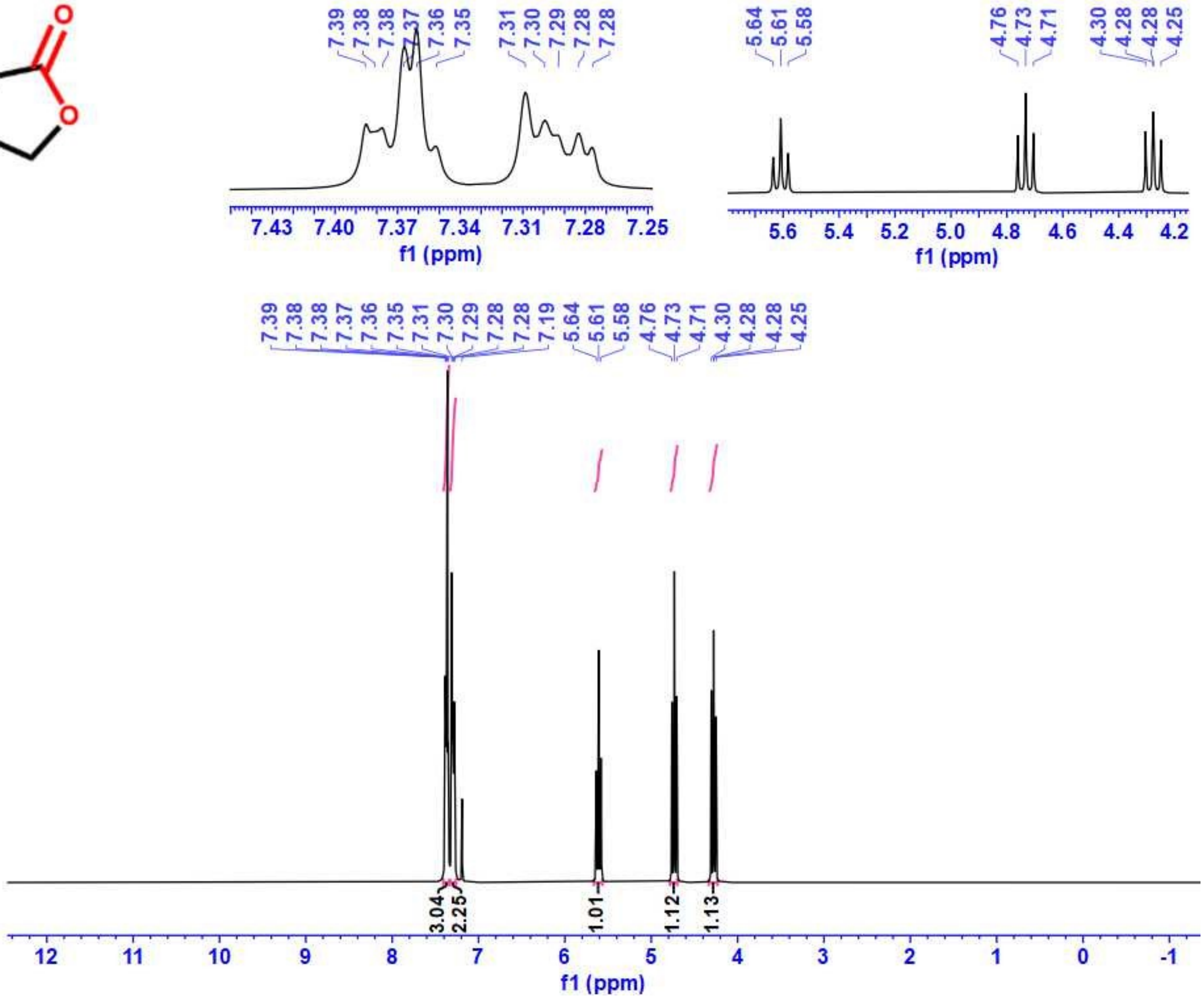

4. ^{13}C NMR spectrum of 4-phenyl-1,3-dioxolan-2-one

^{13}C NMR (75 MHz, $CDCl_3$) δ 71.28, 78.11, 125.99, 129.37, 129.87, 135.90, 154.93.

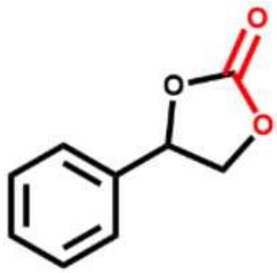

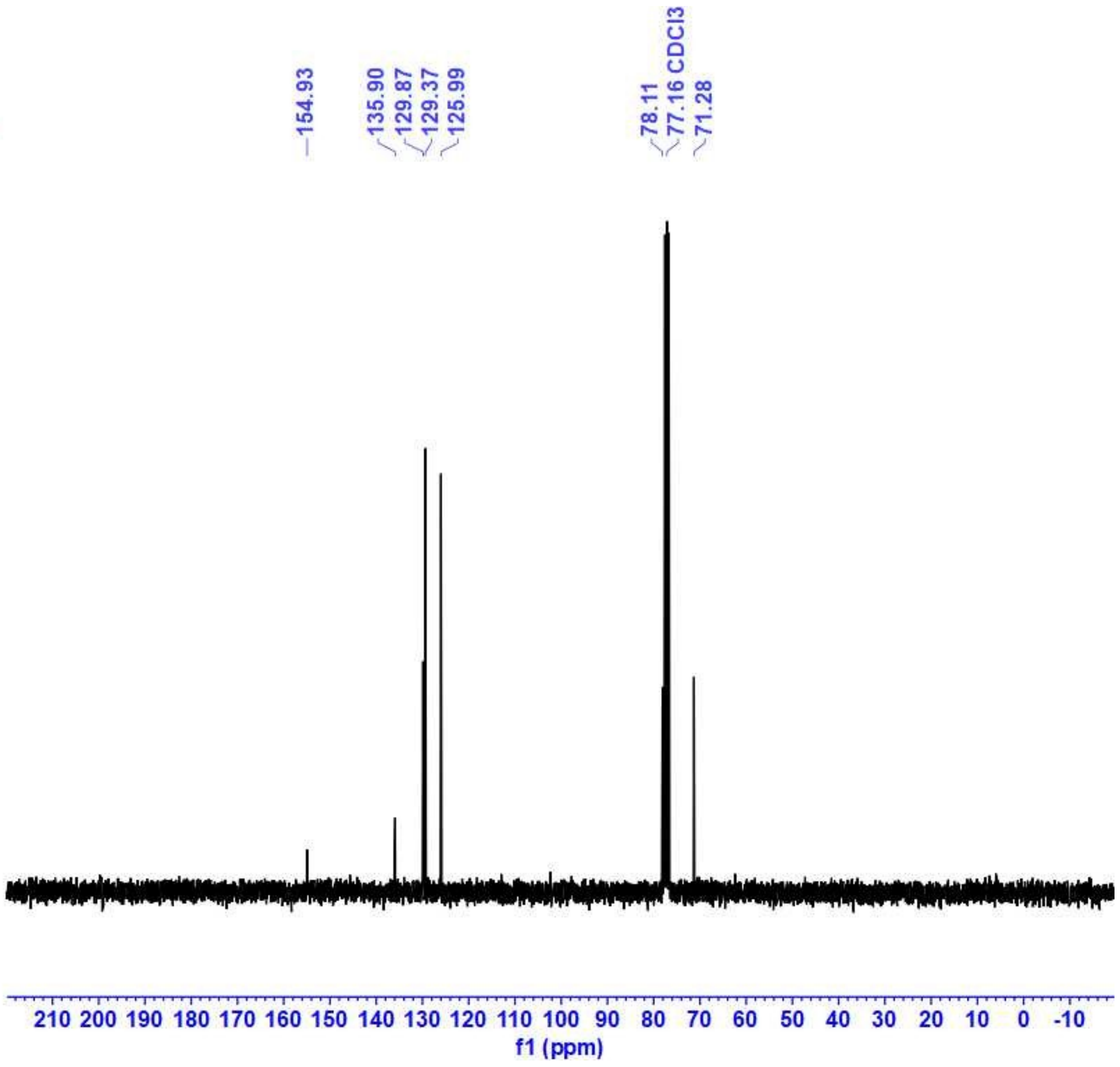

5. ^{1}H NMR spectrum of Hexahydro-1,3-benzodioxol-2-one

^{1}H NMR (300 MHz, $CDCl_3$) δ 1.46 (dq, *J* = 9.4, 5.8 Hz, 2H), 1.68 (dp, *J* = 18.8, 6.7 Hz, 3H), 1.93 (d, *J* = 5.7 Hz, 4H), 4.73 (q, *J* = 4.9 Hz, 2H).

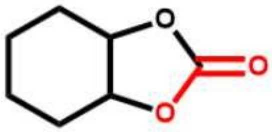

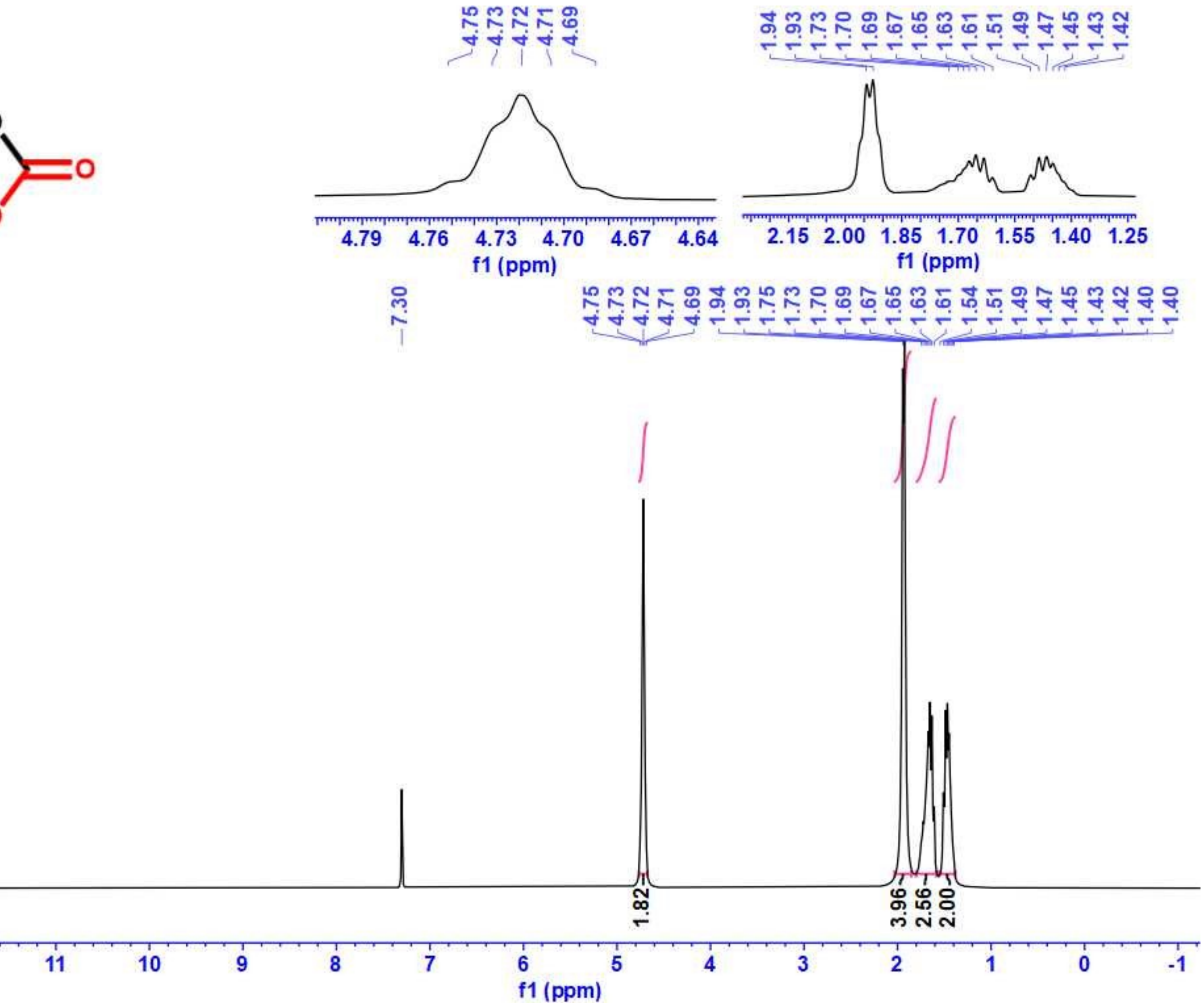

6. ^{13}C NMR spectrum of Hexahydro-1,3-benzodioxol-2-one

^{13}C NMR (75 MHz, $CDCl_3$) δ 19.27, 26.88, 75.84, 155.45.

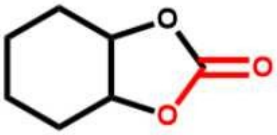

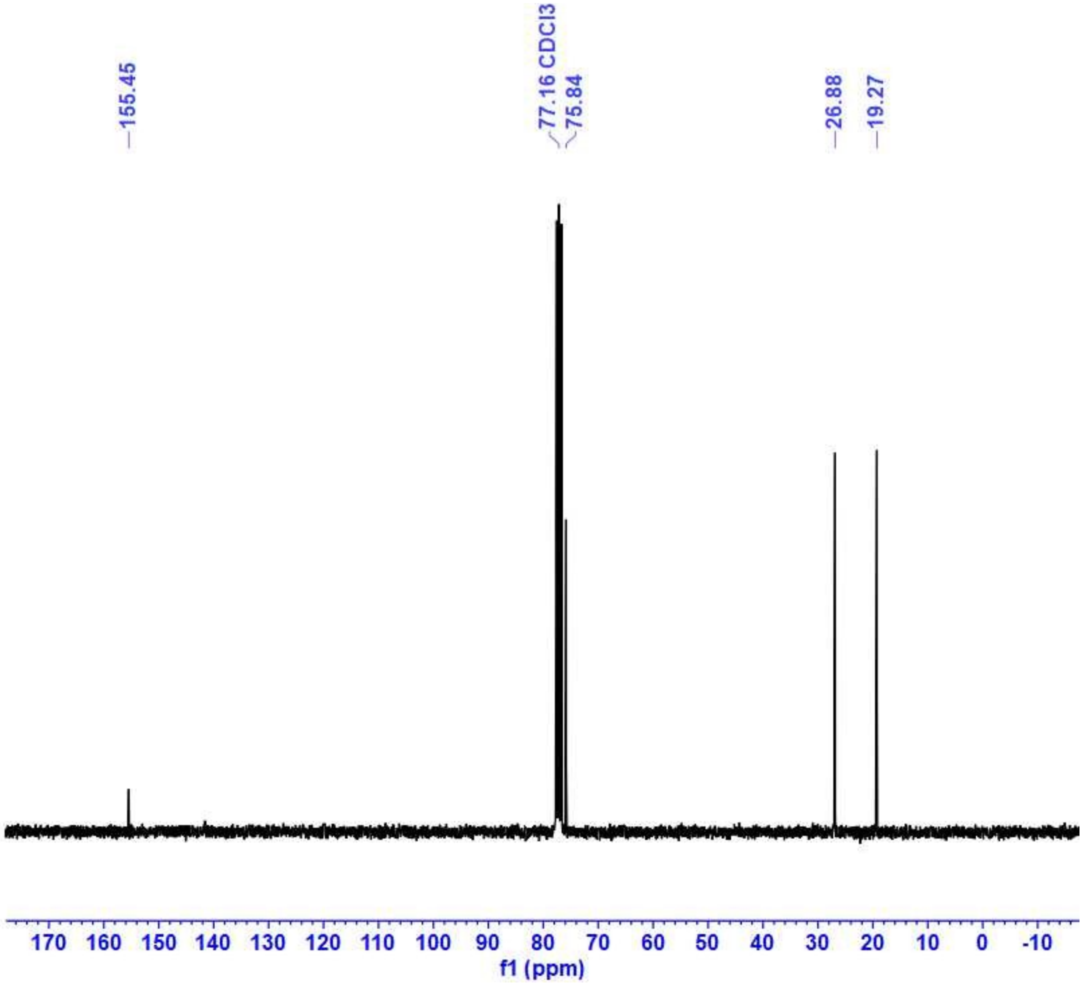

7. ^1H NMR spectrum of 4-ethyl-1,3-dioxolan-2-one

^1H NMR (300 MHz, $CDCl_3$) δ 1.04 (t, *J* = 7.5 Hz, 3H), 1.80 (m, 2H), 4.11 (m, 1H), 4.55 (m, 1H), 4.68 (m, 1H).

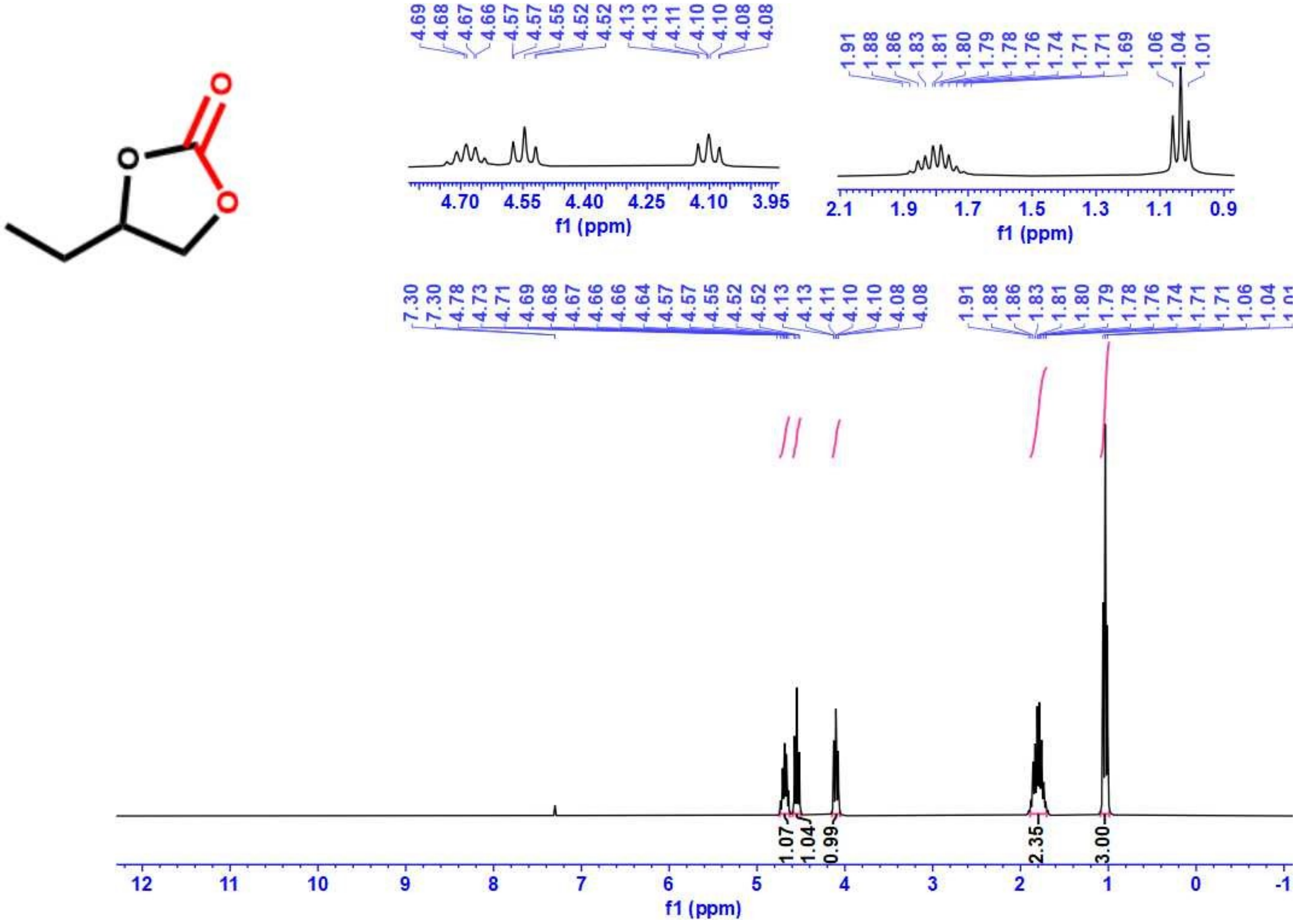

8. ^{13}C NMR spectrum of 4-ethyl-1,3-dioxolan-2-one

^{13}C NMR (75 MHz, $CDCl_3$) δ 8.52, 26.95, 69.10, 78.12, 155.24.

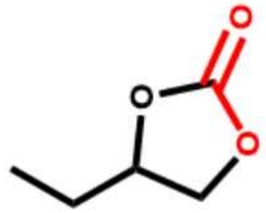

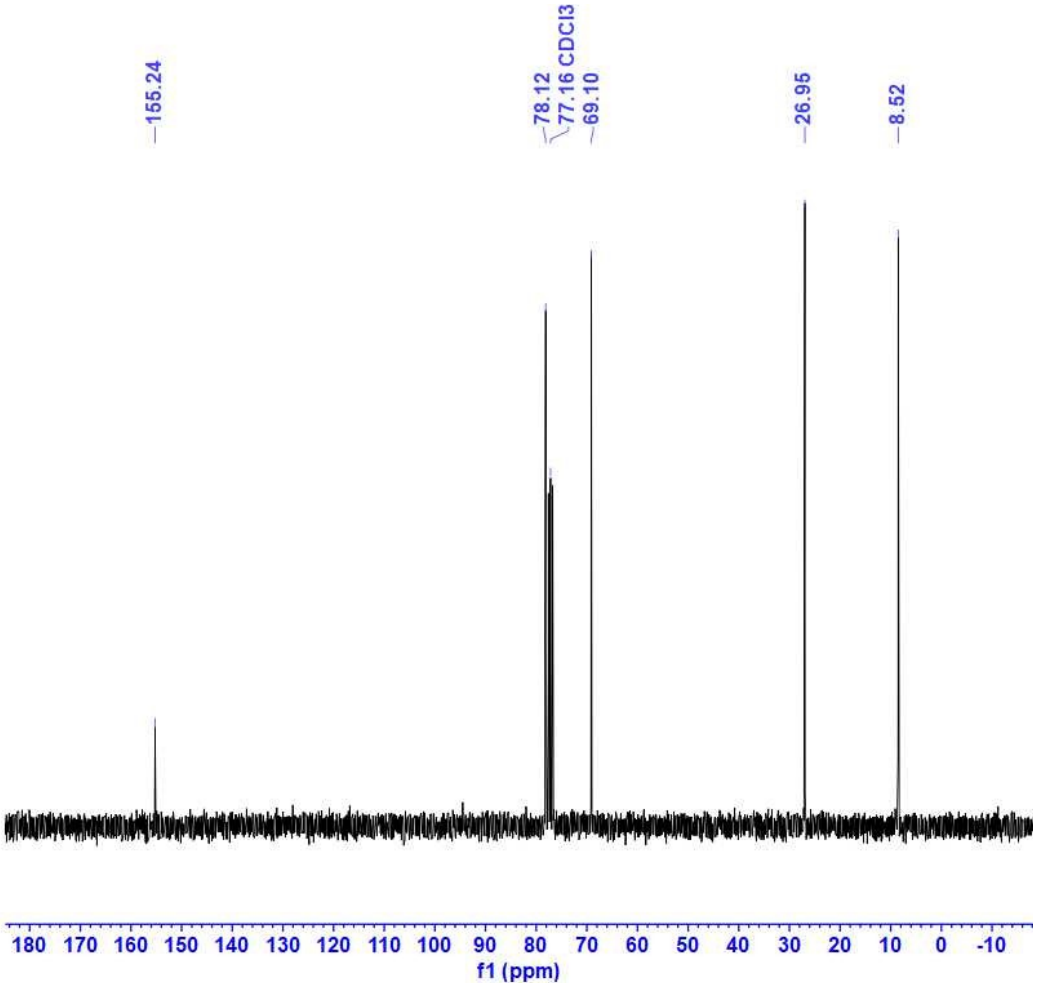

9. ^{1}H NMR spectrum of 4-(phenoxymethyl)-1,3-dioxolan-2-one

^{1}H NMR (300 MHz, $CDCl_3$) δ 4.11 (m, 2H), 4.51 (m, 2H), 4.96 (ddt, *J* = 8.1, 6.0, 3.9 Hz, 1H), 6.84 (m, 2H), 6.95 (m, 1H), 7.24 (m, 2H).

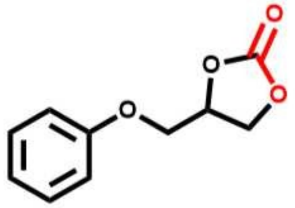

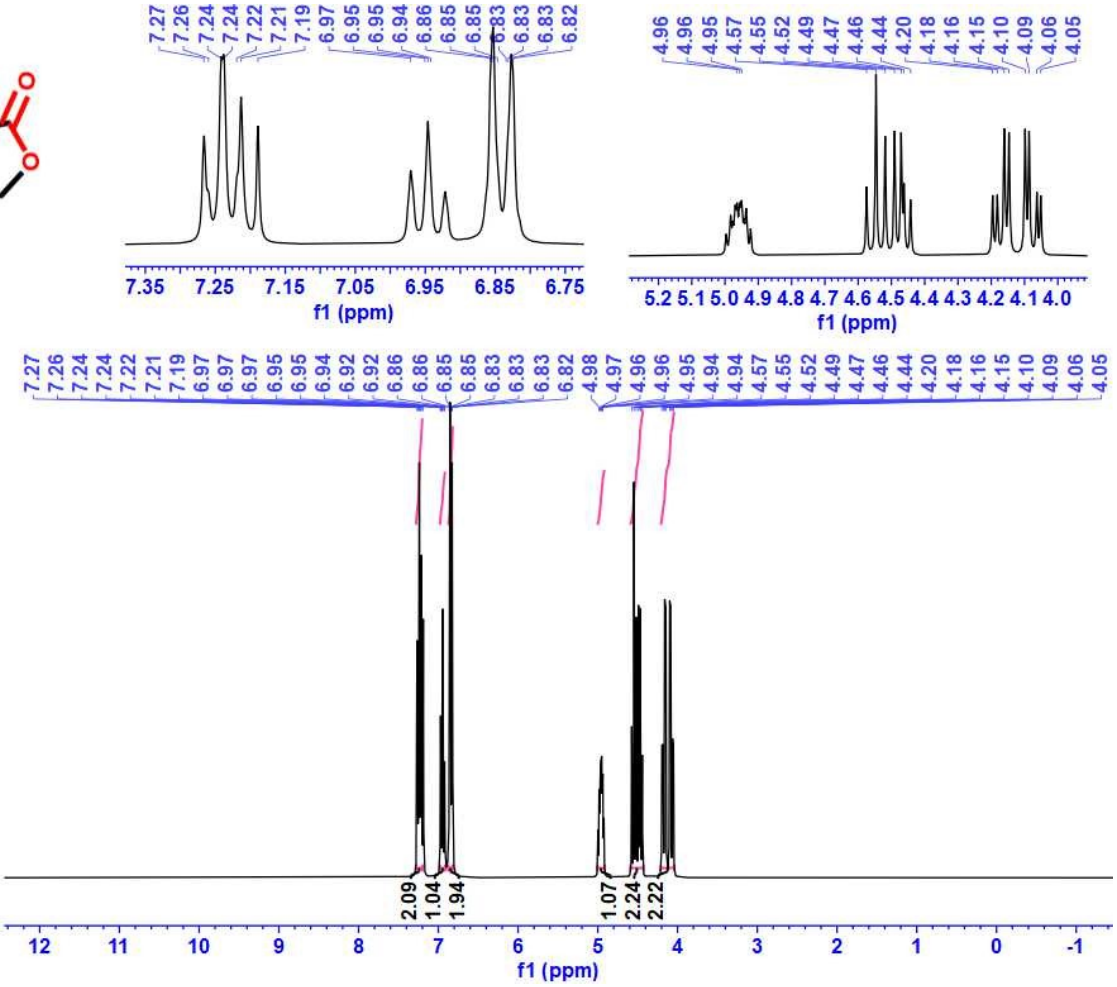

10. ^{13}C NMR spectrum of 4-(phenoxymethyl)-1,3-dioxolan-2-one

^{13}C NMR (75 MHz, $CDCl_3$) δ 66.37, 66.98, 74.22, 114.72, 122.13, 129.83, 154.79, 157.86.

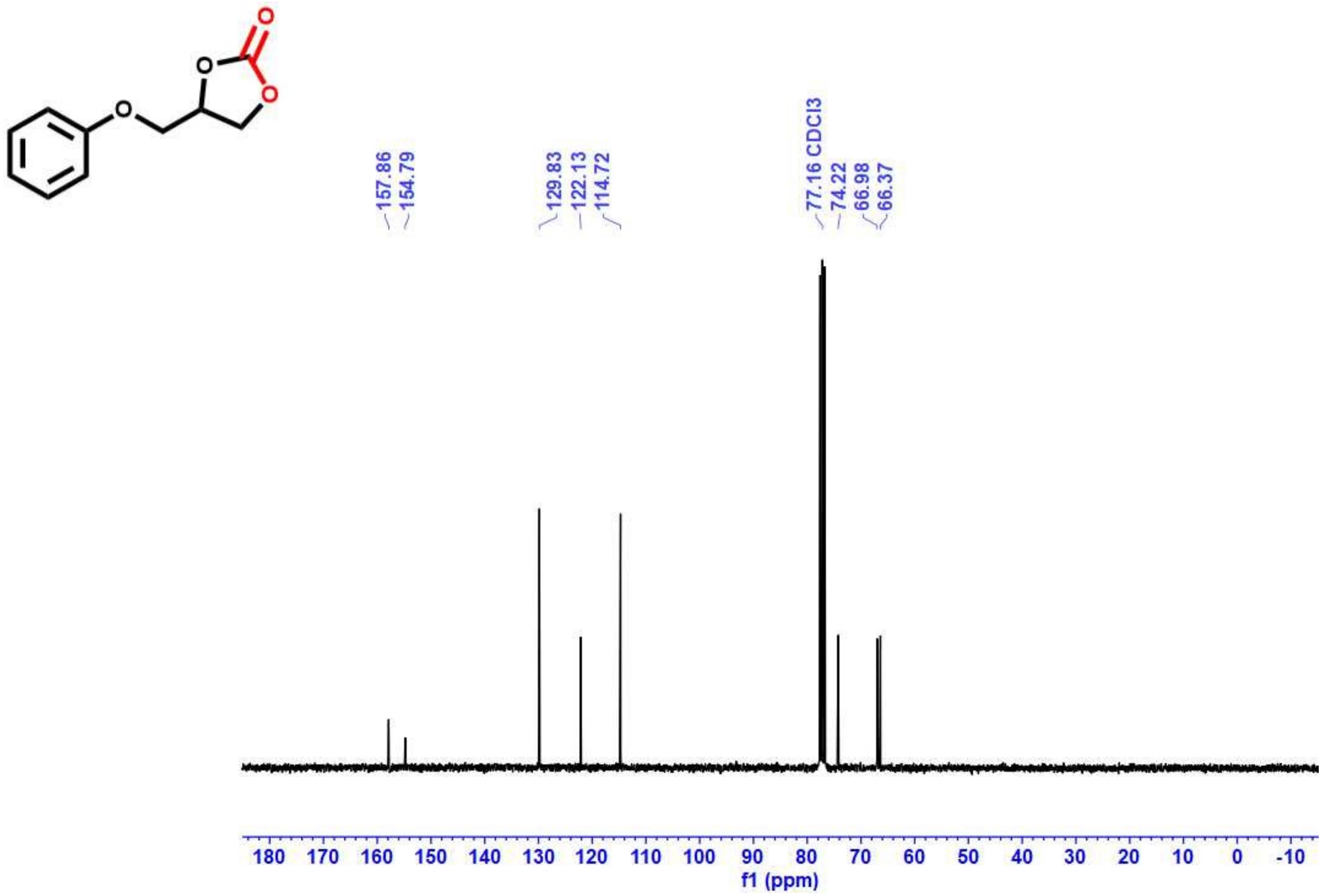

11. ^{1}H NMR spectrum of 4-(Chloromethyl)-1,3-dioxolan-2-one

^{1}H NMR (300 MHz, $CDCl_3$) δ 3.79 (qd, J = 12.4, 4.2 Hz, 2H), 4.51 (m, 2H), 5.02 (m, 1H).

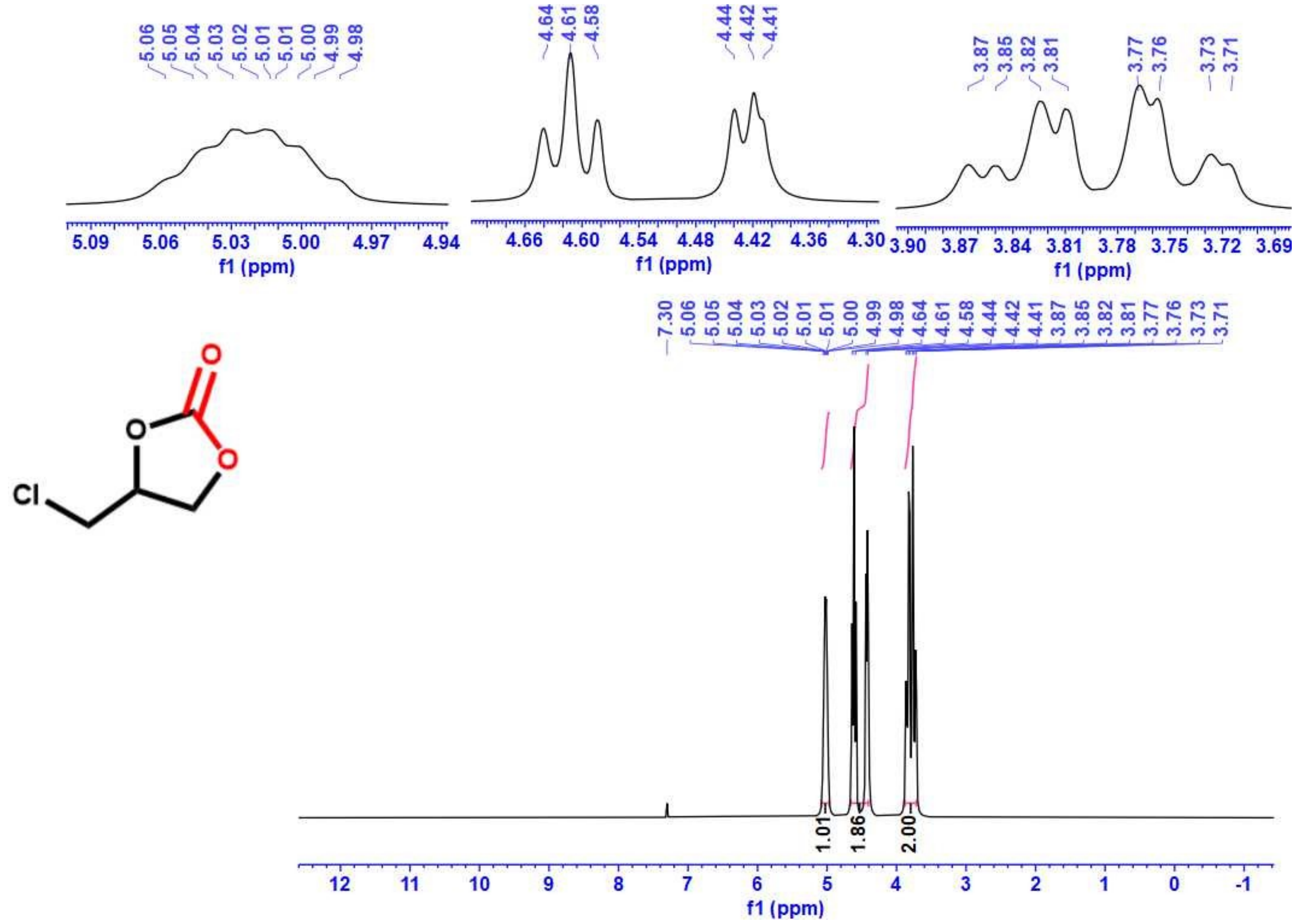

12. ^{13}C NMR spectrum of 4-(Chloromethyl)-1,3-dioxolan-2-one

^{13}C NMR (75 MHz, $CDCl_3$) δ 43.96, 67.01, 74.44, 154.41.

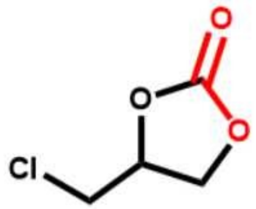

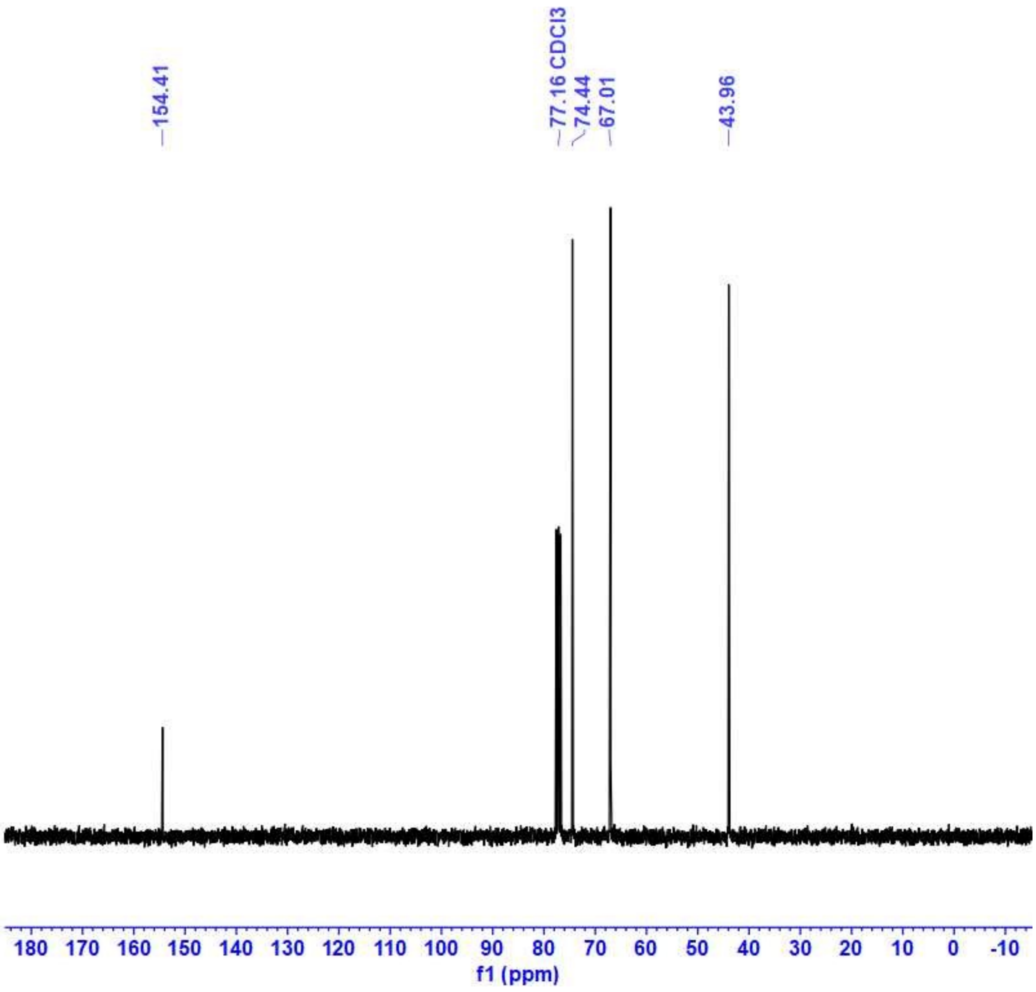

13. ^{1}H NMR spectrum of 4-(isopropoxymethyl)-1,3-dioxolan-2-one

^{1}H NMR (300 MHz, $CDCl_3$) δ 1.18 (d, J = 6.1 Hz, 6H), 3.57 – 3.75 (m, 3H), 4.35 – 4.57 (m, 2H), 4.82 (tdt, J = 7.7, 5.4, 3.2 Hz, 1H).

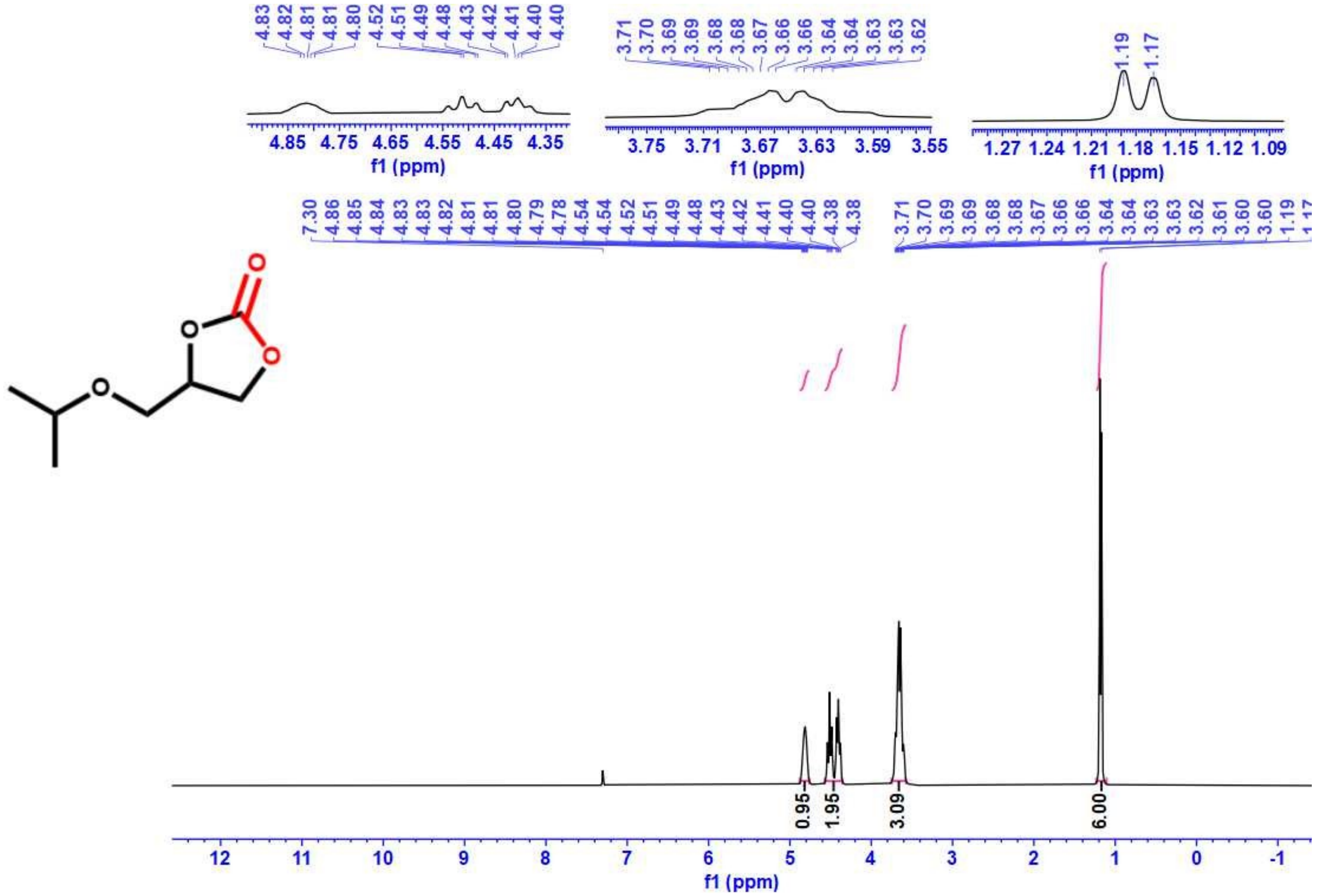

14. ^{13}C NMR spectrum of 4-(isopropoxymethyl)-1,3-dioxolan-2-one

^{13}C NMR (75 MHz, $CDCl_3$) δ 21.87, 66.55, 67.21, 73.04, 75.31, 155.22.

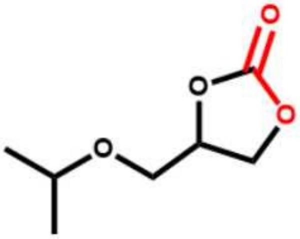

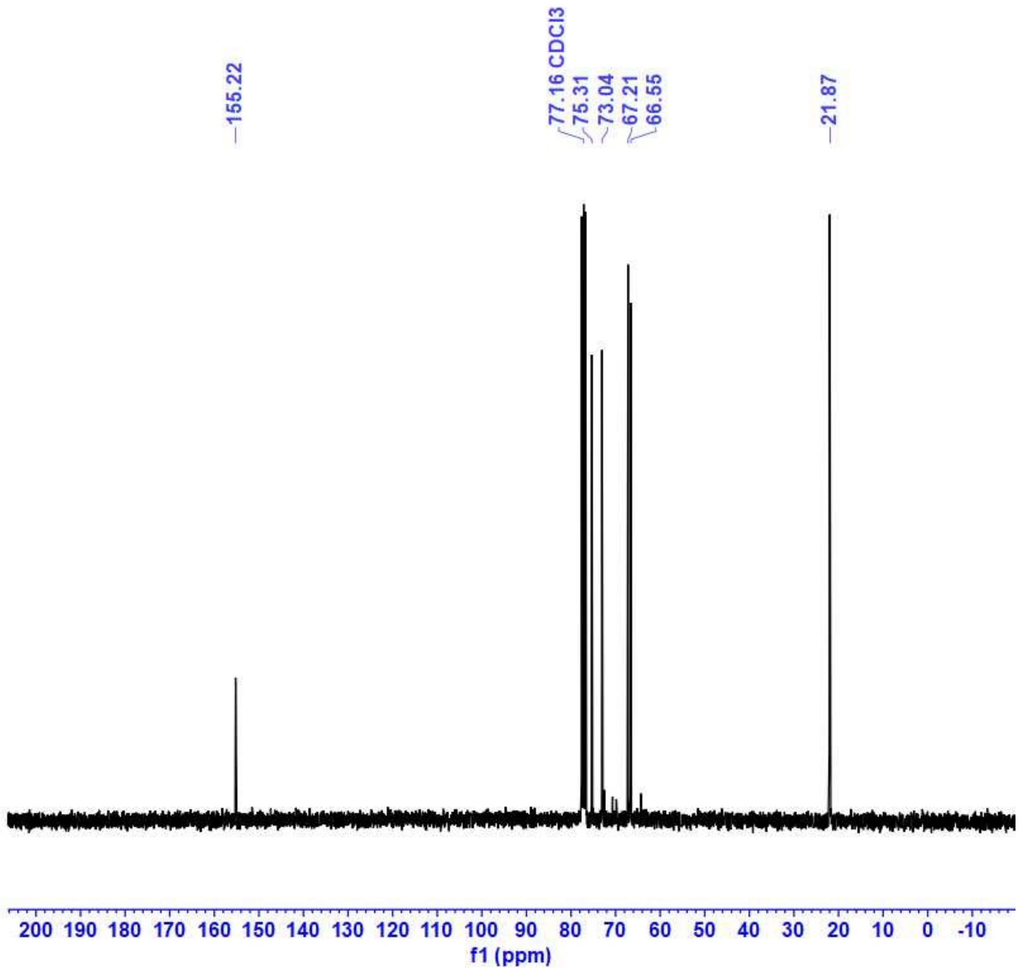

15. ^{1}H NMR spectrum of 4-((allyloxy)methyl)-1,3-dioxolan-2-one

^{1}H NMR (300 MHz, $CDCl_3$) δ 3.66 (m, 2H), 4.05 (m, 2H), 4.46 (m, 2H), 4.84 (ddt, *J* = 7.3, 6.1, 3.7 Hz, 1H), 5.25 (m, 2H), 5.87 (m, 1H).

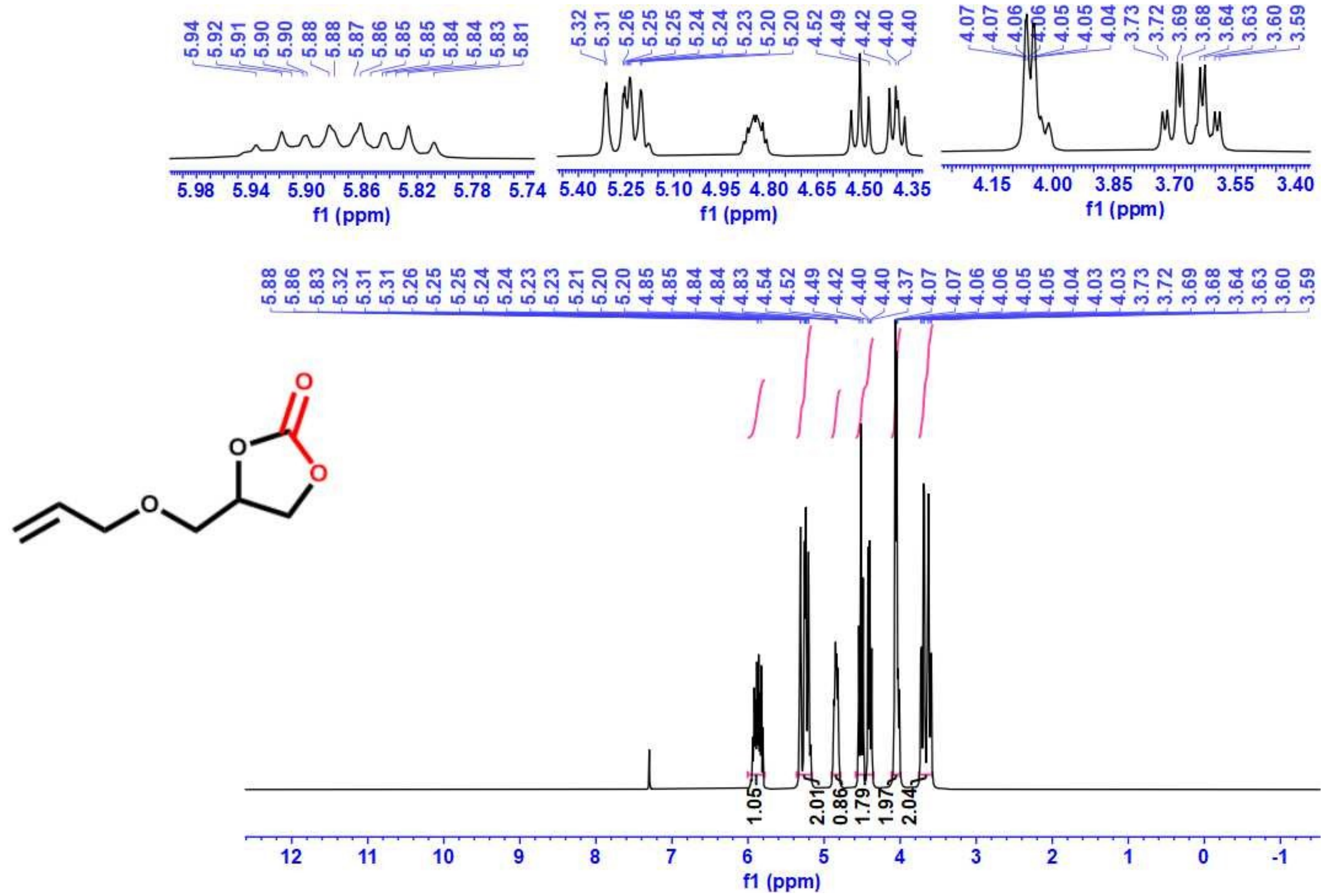

16. ^{13}C NMR spectrum of 4-((allyloxy)methyl)-1,3-dioxolan-2-one

^{13}C NMR (75 MHz, $CDCl_3$) δ 66.31, 68.87, 72.56, 75.18, 117.88, 133.72, 155.10.

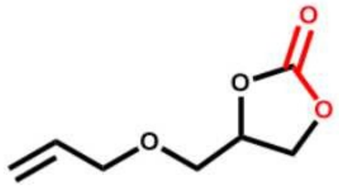

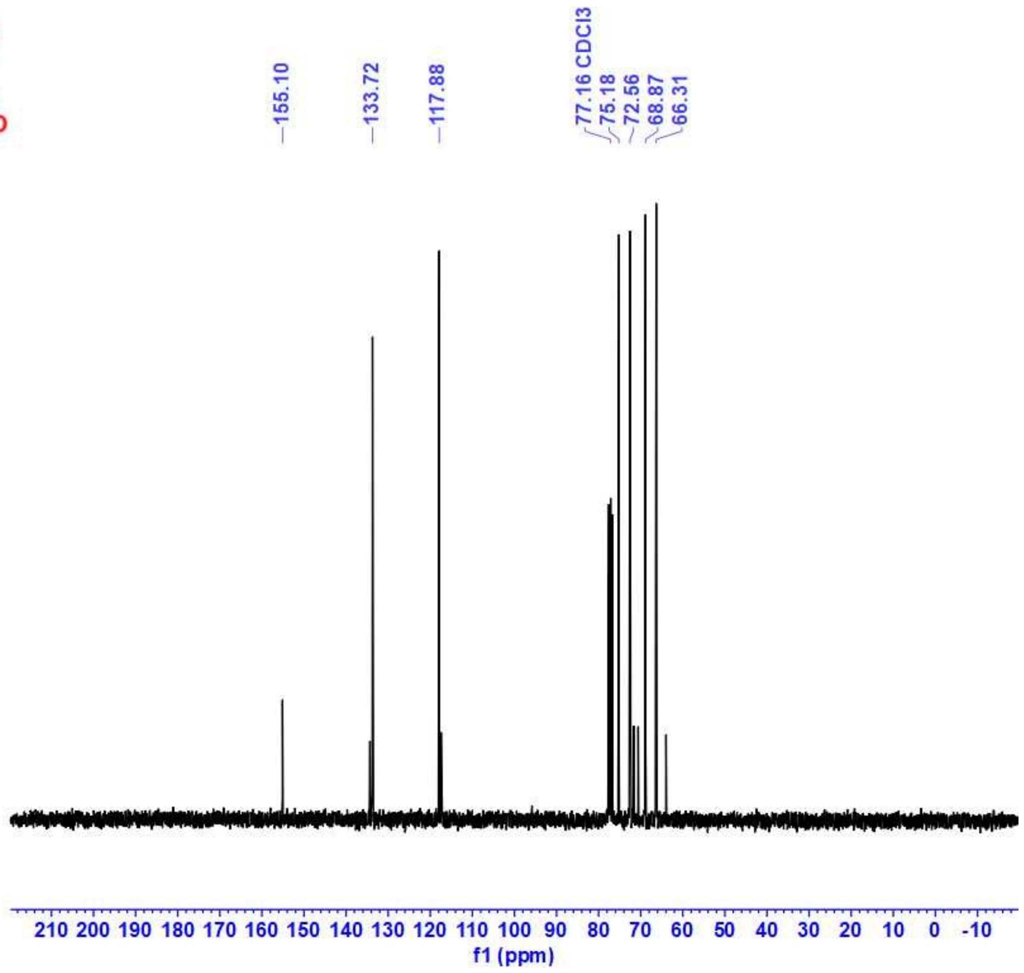

Supplement: RA-015-D4RA08614G-s001 [file RA-015-D4RA08614G-s001.pdf]
